# Neutrophil-related IL1R2 gene predicts the occurrence and early progression of myocardial infarction

**DOI:** 10.3389/fcvm.2025.1516043

**Published:** 2025-03-31

**Authors:** Jieqiong Tang, Xierenayi Tudi, Tianxiang Zhang, Jingbo Zhu, Tongtong Shen

**Affiliations:** ^1^ Department of Cardiology, Chuzhou Hospital Affiliated to Anhui Medical University, Chuzhou, China; ^2^ Beijing Institute of Heart, Lung and Blood Vessel Diseases, Beijing Anzhen Hospital, Capital Medical University, Beijing, China; ^3^ Department of Cardiology, Ruijin Hospital, School of Medicine, Shanghai Jiao Tong University, Shanghai, China; ^4^ Department of Urology, Renji Hospital, School of Medicine, Shanghai Jiao Tong University, Shanghai, China; ^5^ Department of Cardiology, Union Hospital, Tongji Medical College, Huazhong University of Science and Technology, Wuhan, China

**Keywords:** neutrophil, diagnosis, early progression, myocardial infarction, mouse model, single cell RNA analysis

## Abstract

**Introduction:**

Myocardial infarction (MI) is a leading cause of death worldwide. Immune cells play a significant role in the MI development. This study aims to identify a marker related to neutrophil for the diagnosis and early progression of MI.

**Methods:**

Key genes were screened using three machine learning algorithms to establish a diagnostic model. A gene associated with the early progression of MI was identified based on single cell RNA sequencing data. To further validate the predictive value of the gene, the mouse models of MI were constructed. Immunofluorescence (IF) analysis demonstrated the co-expression of the gene with neutrophils. Immunohistochemistry (IHC) was performed to validate the role of the gene in the progression of MI.

**Results:**

Neutrophils were identified and verified as the key infiltrating immune cells (IICs) involved in the onset of MI. A diagnostic panel with superior performance was developed using five key genes related to neutrophils in MI (AUC = 0.887). Among the panel, IL1R2 was found to early phase of MI, which was further corroborated by IHC in mouse models of MI.

**Conclusions:**

This study suggests that IL1R2, which is specific to neutrophils, can predict the diagnosis and early progression of MI, providing new insights into the clinical management of MI.

## 1 Introduction

A common consequence of coronary heart disease, myocardial infarction (MI), has high morbidity and mortality rates, making it a detriment to global health (1). MI is characterized by extensive myocardial damage and dysfunction caused by an abrupt blockage of the bloodstream (2). There are several risk factors for MI including smoking, alcohol intake, hypertension, dyslipidemia, and diabetes mellitus (3). In recent years, with the development of serum biomarkers, electrocardiography, and interventional therapies, survival rates of MI have increased by 15% (4). Serum markers of MI commonly used in clinical diagnosis include cardiac troponin T (cTnT) (5), cardiac troponin I (cTnI), cardiac myoglobin, and creatine kinase-MB (CK-MB) (6, 7). However, increased blood concentrations of cTnT only indicates myocardial damage induced by ischemia and hypoxia and not abnormal perfusion. Meanwhile, the sensitivity and specificity of known markers are limited because increased levels of the markers are found in other diseases such as heart and renal failure (8). Simultaneously, revascularization can cause reperfusion injury which contributes to up to 50 percent of post-infarction sequelae (9). Furthermore, although approximately 90 percent of patients have experienced chest pain, this discomfort was not indicative of MI (10). Some patients with MI may not display evident symptoms, and noticeable electrocardiogram (ECG) alterations may not be present. Furthermore, potential biomarkers related to specific molecular function have been identified for MI diagnosis (11). Therefore, the identification of novel biomarkers and improvement of early-MI diagnostic model efficiency are urgently needed.

Several studies have shown that immune cells play a critical role in MI. Peripheral blood contains several types of immune cells including neutrophils, lymphocytes, and macrophages. Many studies have confirmed that neutrophils play a significant role in inflammatory reactions and heart repair (12). Single cell RNA sequencing (scRNA-seq) has improved the method to study the relationship between immune cells and progression of illness. ScRNA-seq can classify sequencing data to reveal difference in cell subpopulations and their proportions (13). In our study, the cell types related to pathogenesis of myocardial infarction were identified by scRNA-seq technology. With the aggravation of myocardial infarction, changes in the ratio of each cell type were quantified using scRNA-seq. Immunofluorescence and immunocytochemistry were conducted to reveal the co-expression of hub gene with key infiltrating immune cells (IICs) and the progression of MI. Although gene tests can be accomplished with 15 min as modern research advances, which can reduce the time to diagnosis of MI, we have yet to identify a gene with great specificity (14).

This study aims to identify a biomarker to predict the clinical diagnosis and early progression of MI. To explore the relationship between neutrophils and MI risk, we downloaded two microarray datasets (GSE66360 and GSE48060) and a scRNA-seq dataset (GSE163465) from the Gene Expression Omnibus (GEO) database. Through a comprehensive analysis of the immune microenvironment in MI, we identified neutrophils as key immune cells that play a central role in the onset of MI. Three machine learning techniques were employed to identify key genes predictive of MI for constructing a diagnostic panel. Subsequently, scRNA-seq was used to unveil the changes of neutrophil ratio and expression of IL1R2 in neutrophil and all cells in all MI periods. Followed by construction mouse models, IL1R2 was validated as a neutrophil-specific marker through immunofluorescence. Finally, the progression of MI was displayed with immunocytochemistry.

## 2 Materials and methods

### 2.1 Dataset collection

**Figure 1:**
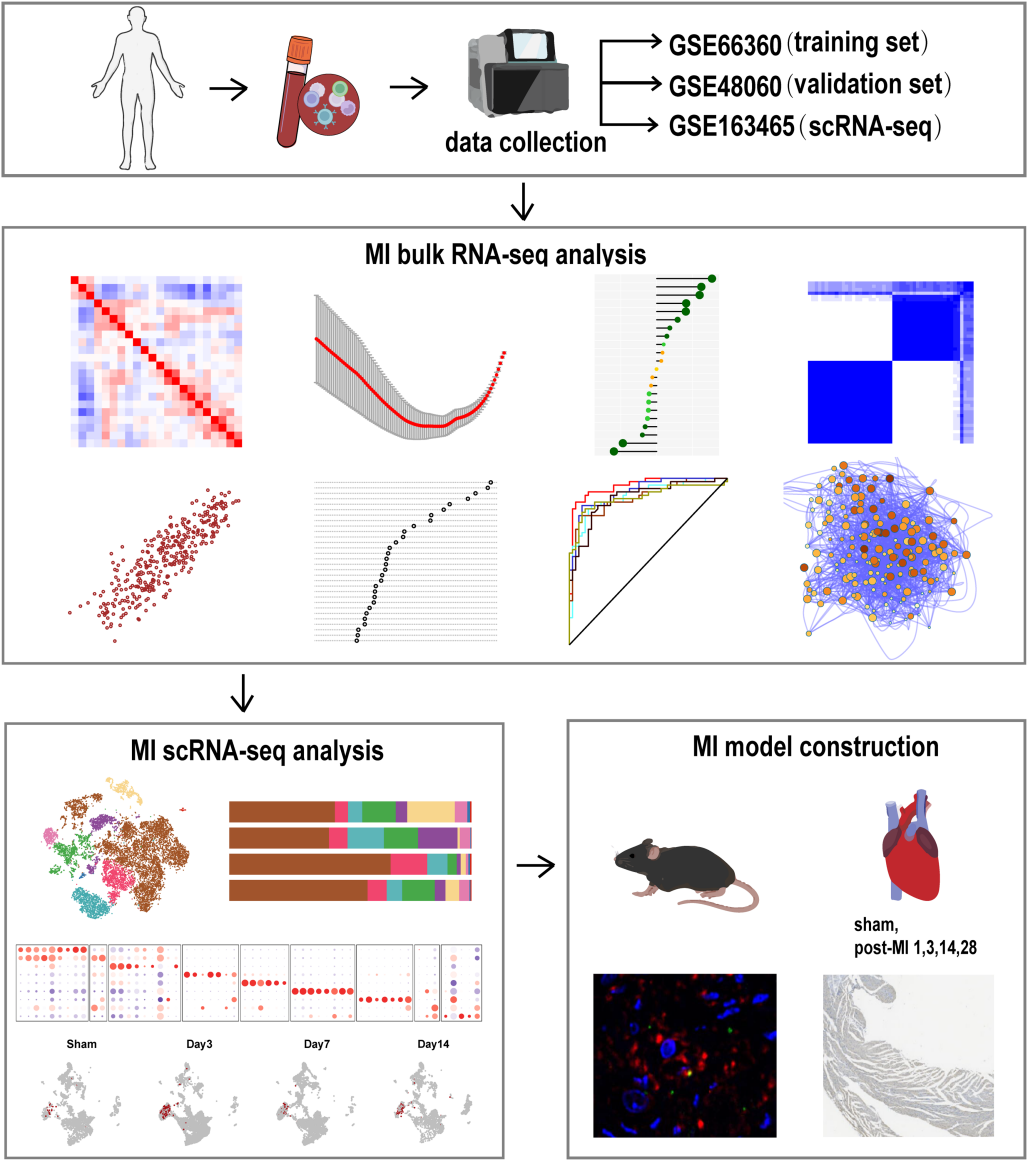
A flow chart of the study protocol is shown.

Three datasets were used to evaluate regulators of MI: GSE66360, GSE48060 and GSE163465. The GSE66360 dataset was downloaded from the GEO database (http://www.nbi.nlm.nih.gov/geo/) and included 49 MI and 50 healthy control samples. The dataset was used to identify genes differentially expressed in MI. The GSE48060, including 31 MI and 21 healthy control samples, and GSE163465, a scRNA-seq dataset of mice, were also obtained from the GEO database and used as validation sets. The normalizeBetweenArrays function from the “limma” package was used to normalize all raw expression data. Gene expression values determined from datasets were transformed into log2(X + 1) counts of reads. An overview of the design of the study is presented in Figure 1.

### 2.2 CIBERSORT

“CIBERSORT,” a comprehensive algorithm, was used to calculate relative proportions of IIC subtypes based on normalized gene expression data. IICs include *T* cells, B cells, macrophages, dendritic cells (DCs), natural killer (NK) cells, monocytes, mast cells, eosinophils, and neutrophils. Subsequently, the “corrplot” package was used to build a heatmap for visualizing correlations among IICs, while the “vioplot” package was used to draw violin plots comparing IIC-related differences between MI and healthy samples using GSE66360 and GSE48060 data. IICs identified with a confidence level of *p* < 0.05 in both datasets were selected for further analysis.

### 2.3 Identification of DEGs

Genes differentially expressed between MI and healthy samples in the GSE66360 dataset were identified using the “limma” package in R. Fold changes (FCs) of each gene expression level were calculated. Genes with |logFC| > 0.5 and adjusted *p* < 0.05 values were considered differentially expressed genes (DEGs). The “EnhancedVolcano” package was used to generate volcano plots. The “pheatmap” package was used to obtain a heatmap based on DEG data identified.

### 2.4 WGCNA analysis

The “WGCNA” package of R was used to construct a gene co-expression network based on identified DEGs. First, any outliers were assessed using “goodSamplesGenes” of the “WGCNA” package. Outliers were removed after setting the cut height. Secondly, the best soft thresholding power (*β* = 6) was screened using the R function, pickSoftThreshold, to build an unsigned network. To ensure network nonscaling, the adjacency matrix was transformed into a topological overlap matrix (TOM). Subsequently, genes with similar expression patterns were grouped into independent modules via hierarchical clustering (minModuleSize = 30, deepSplit = 2, mergeCutHeight = 0.15). A heatmap was drawn to describe the relationship between co-expression modules and the infiltration fractions of key immune cells. Furthermore, we analyzed the correlation between the key module and key IICs using gene significance (GS) and module membership (MM). Resultant data were visualized using the Cytoscape (version 3.7.1) network of module eigengenes. Genes with GS > 0.6 and MM > 0.7 in the significant module were identified as key genes. Then, the “NetworkAnalyzer” function in Cytoscape was utilized to calculate node degree, with nodes >300 degrees in the WGCNA network selected for further analysis. Finally, hub genes were identified by intersecting key genes with selected nodes. A web tool (http://bioinformatics.psb.ugent.be/webtools/Venn/) was used to create a Venn diagram for identifying intersecting genes.

### 2.5 Functional enrichment analysis

To investigate the biological functions of the key modules, the R package “clusterProfiler” was used to perform Gene Ontology (GO) and Kyoto Encyclopedia of Genes and Genomes (KEGG) functional enrichment analyses. GO analysis included three aspects: biological processes (BP), cell composition (CC), and molecular function (MF). The “treemap” package was used for visualizing KEGG function analysis, and the “GOplot” package was used to diagram GO-related data. GO term and KEGG pathways with adjusted *p*-values < 0.05 were considered statistically significant. Metascape (http://metascape.org) is an emerging algorithm used for investigating biological processes underlying transcriptome data and related signaling pathways. Protein-protein interaction (PPI) analysis was performed in the key module using Metascape. Function and hub clusters of the PPI network were visualized using Cytoscape (version 3.7.1).

### 2.6 Identification of hub genes using machine learning techniques

Key genes were identified using three machine learning algorithms. To avoid the overfitting of intersecting genes, the “glmnet” package was used to construct a least absolute shrinkage and selection operator (LASSO) binomial logistic regression. Then, support vector machine recursive feature elimination (SVM-RFE) was performed to shrink the feature set and minimize cross-validation error when identifying key genes suing “e1071”, “kernlab” and “caret” packages. A random forest algorithm was used to rank genes using the “randomForest” package, with a threshold set to MeanDecreaseGini score >2 (ntree = 600). Finally, a Venn diagram was used to visualize intersections between genes identified via the three methods (LASSO, SVM-RFE, and random forest).

### 2.7 Evaluation of hub genes

GSE48060, containing 31 MI and 21 healthy control peripheral blood samples, was used to verify expression patterns of hub genes. To compare expression levels of key genes expressed in MI and healthy control samples, box plots were drawn using the “ggpubr” package. Then, the predictive accuracy of hub genes was evaluated via receiver operating characteristic (ROC) curves using the R package “pROC”. In addition, decision curve analysis (DCA) was conducted to evaluate net benefit of each gene for predicting MI at every probability threshold using the “rmda” package.

### 2.8 Construction and validation of a predictive nomogram

A nomogram integrating several diagnostic determinants was constructed to improve the diagnosis of MI. The “rms” package was used to build a nomogram based on genes identified via machine learning algorithms to evaluate the risk of suffering from MI. Harrell's concordance index (C-index) and area under the curve (AUC) assessments were applied to reveal the predictive ability of the nomogram, with a C-index >0.9 considered highly accurate. Meanwhile, a calibration plot comparing differences between predictions and actual observations was drawn to evaluate the performance of the diagnostic model using the “rms” package. Furthermore, we used GSE48060 to validate the accuracy of the nomogram model.

### 2.9 Establishing the risk model

The regression coefficient was calculated via logistic regression using the “rms” package, with the risk score determined, as follows:

$$\mathrm{Risk}\;\mathrm{score}=\sum_{i\;=\;1}^{n}\mathrm{Coe}f_{i}\times \mathrm{Ex}p_{i},$$

where Coef represents the regression coefficient of each hub gene and Exp represents the expression level of every gene.

### 2.10 Correlation between hub genes and IICs

Spearman's correlation analysis was used to reveal the relationship between key genes and IICs. Then, a scatter plot was generated using “ggpubr” and “ggExtra” packages in RStudio to visualize results.

### 2.11 Gene set enrichment analysis (GSEA)

GSEA (http://software.broadinstitute.org/gsea/index.jsp) was used to identify significantly related signaling pathways that were enriched in both MI and healthy control groups. The annotated gene set list was selected from c2.cp.kegg.v7.2.symbols.gmt as the reference gene set. After performing 1,000 permutations, cut-off criteria of enriched gene sets were set to a false discovery rate (FDR) q-value < 0.25 and nominal *p*-value < 0.05. GSEA (version 4.3.1) was used to screen for signaling pathways associated with high-risk patients with MI. Then, the “clusterProfiler” package of R was used to assess signaling pathways enriched in low-risk patients with MI.

### 2.12 Identification of MiRNAs and construction of a CeRNA network

MiRNAs potentially binding to key genes were predicted using four databases: miRcode (http://www.mircode.org/), miRWalk (http://mirwalk.umm.uni-heidelberg.de/), MicroT-CDs (http://diana.imis.athena-innovation.gr/DianaTools/index.php?r=MicroT_CDS/index), TarBase v7.0 (http://diana.imis.athena-innovation.gr/DianaTools/index.php?r=tarbase/index). A Venn diagram is used to display the overlap between the prediction results. The miRNAs that appeared in more than one database were identified as key miRNAs. Finally, the key miRNAs and genes were used to construct a ceRNA network using Cytoscape (version 3.7.1).

### 2.13 Consensus clustering analysis

Based on expression levels of key genes in samples of patients with MI, consensus clustering was applied to stratify patients into discrete subgroups. The number of subtypes ranged from two to nine. The unsupervised clustering “km” method based on Euclidean distance was repeated 50 times to guarantee the stability and accuracy of subtypes using the “ConsensusClusterPlus” package of R. The sample distribution of each cluster was revealed using principal component analysis (PCA) and t-SNE method. Subsequently, subtype-specific expression levels of hub genes were visualized using boxplots and heatmaps created with “ggplot2”, “ggpubr”, “pheatmap” packages. Finally, the “PupillometryR” package was utilized to calculate neutrophil infiltration fractions, immune scores, stromal scores, and estimate scores of the subtypes, with the data displayed using violin plots.

### 2.14 Gene set variation analysis (GSVA)

GSVA was applied to estimate the enrichment of key signaling pathways via a non-parametric and unsupervised method. In this study, we divided patients with MI into two groups based on genes related to neutrophils. The GSVA package in R was used to comprehensively score all gene sets and analyze the differences in biological functions between two subtypes.

### 2.15 scRNA-Seq analysis

The “Seurat” package was used to perform steps including filtering samples, identifying normalized highly variable genes, reducing dimensionality and clustering cells. The cells expressing more than 200 genes, fewer than 2,500 genes, and genes expressed in more than 3 cells were selected to remove low-quality cells and possible doublets. Then, the highly variable genes were identified with FindVariableFeature function after normalizing data. Uniform manifold approximation and projection (UMAP) and t-Distributed stochastic neighbor embedding (t-SNE) were used to scale down the dimension of all genes. Subsequently, the FindNeighbors and FindClusters functions were conducted to determine the subgroups of cells followed by annotating manually. The expression of hub genes in each cell subgroup was calculated using R package “scCustomize”.

### 2.16 Mice and myocardial infarction model

According to previous research, the progression of ischemic cardiomyopathy is slowed down by estrogen, wild-type male C57BL/6 mice which were eight weeks old were used in our study (15, 16). The mice provided by Shanghai JieSiJie Laboratory Animal Co., Ltd were divided into four groups at random: sham group, 1d post-MI group, 3d post-MI group, 14d post-MI group. With the approval of the Animal Care Committee of Ruijin hospital, all animal experimental procedures were conducted.

Permanent left anterior descending artery ligation was conducted to construct MI models. Briefly, the mice anesthetized with 1.5% isoflurane were intubated with 22-gauge tubes and placed on a heating pad to maintain the body temperature at 37°C. Then a horizontal incision between 3rd and 4th intercostal spaces was made on the chest to expose the heart after disinfection. An 8-0 silk suture was used to ligate the left anterior descending (LAD) artery and 5-0 and 3-0 silk sutures were utilized to close the chest and skin. Sham-operated mice involved the same procedures except coronary artery ligation.

### 2.17 Western blotting (WB)

Total proteins were extracted from mouse heart tissue and the protein concentration was determined using a BCA (Bicinchoninic Acid) assay. Equal amounts of protein were separated by sodium dodecyl sulfate-polyacrylamide gel electrophoresis (SDS–PAGE, 7.5%–12% gels). Then the proteins were transferred to polyvinylidene fluoride (PVDF) membranes. The membrane was incubated overnight with the an anti-Il1r2 antibody (1:3000, AF06912, AiFang, Shanghai, China) after blocking the membrane with 5% bovine serum albumin (BSA). Following washing, the membrane was incubated with a goat anti-rabbit secondary antibody at room temperature for 1 h. Protein bands were visualized using a Luminescent Image Analyzer detection system (Fujifilm, LAS-4000). The level of Gapdh expression (1:20000, HRP-6004, Proteintech) was used as an internal control.

### 2.18 Quantitative polymerase chain reaction (qPCR)

Total RNA was extracted from heart tissue of myocardial infarction (MI) mice using TRIzol reagent (Invitrogen, Carlsbad, CA, USA). Then, cDNA synthesis was performed using PrimeScriptTM RT reagent kit (Cat# 4368813, Thermo Fisher Scientific). Quantitative polymerase chain reaction (qPCR) analysis run in the Opticon Real-time PCR Detection System (Bio-Rad) using SYBR Green master mix (Toyobo, Japan). We used Primer software to design IL1R2, GAPDH primer. IL1R2: Forward: 5’-TCAGGAAGTTGGTGCGGACAATG-3’ and reverse: 5’-TGTCGGAGTGAGGTGCCAAGG-3’. GAPDH: Forward: 5’-CAG-GGC-TGC-TTT-TAA-CTC-TGG-TAA-3’ and reverse: 5’- GGGTGG-AAT-CAT-ATT-GGA-ACA-TGT-3’. The relative mRNA expression was normalized to GAPDH expression and quantified using the comparative Ct (*ΔΔ*Ct) method.

### 2.19 Immunofluorescence

The paraffin sections of the mouse heart were used for immunofluorescence to probe the expression of IL1R2. The 4% paraformaldehyde (PFA) was conducted to fix the indicated hearts at 4°C for 2 h after euthanizing the mice before MI, on the first, third and fourteenth day after MI. The hearts were buffered with 30% sucrose for 4 h after cryoprotection with 20% sucrose. Then, through deparaffinization, hydration, antigen retrieval with pH8.0 Tris-EDTA, the sections were blocked with 3% hydrogen peroxide for 15 min at room temperature and incubated with anti-Il1r2 (1:3000, AF06912, AiFang, Shanghai, China) and anti-MPO (1:3000, Ab208670, Abcam, Shanghai, China) antibodies at 4°C overnight. The phosphate-buffered saline was utilized to wash the sections followed by staining with CD31HRP-Polymer anti-rabbit IgG for 30 min at room temperature. Finally, the nuclei of mouse heart section was stained with DAPI for 10 min.

### 2.20 Immunohistochemistry

The heart cells were seeded in the culture plates. After washing the cells three times with PBS at room temperature for 30 min, the cells were permeabilized using 0.1% T riton X-100 (Beyotime Biotechnology, Shanghai, China) for 15 min at 37°C. Subsequently, antibodies mentioned earlier were used to incubate the cells as immunofluorescence. Finally, the QuPath software (v0.5.0) was conducted to calculate the percentage of positive cells.

### 2.21 Statistical analysis

R software (version 4.2.1, https://www.rstudio.com/) was used to perform data analyses and visualization and Prism 7.0 was used to visualize data comparing models of four articles. PCA was performed to reduce the dimensionality and identify a pattern based on GSE66360 data using the “scatterplot3d” package. The Student's *t*-test was used to compare paired data; for example, those of MI and healthy control datasets. For all analyses, values of *p* < 0.05 were considered statistically significant.

## 3 Results

### 3.1 Screening for Key IICs

**Figure 2:**
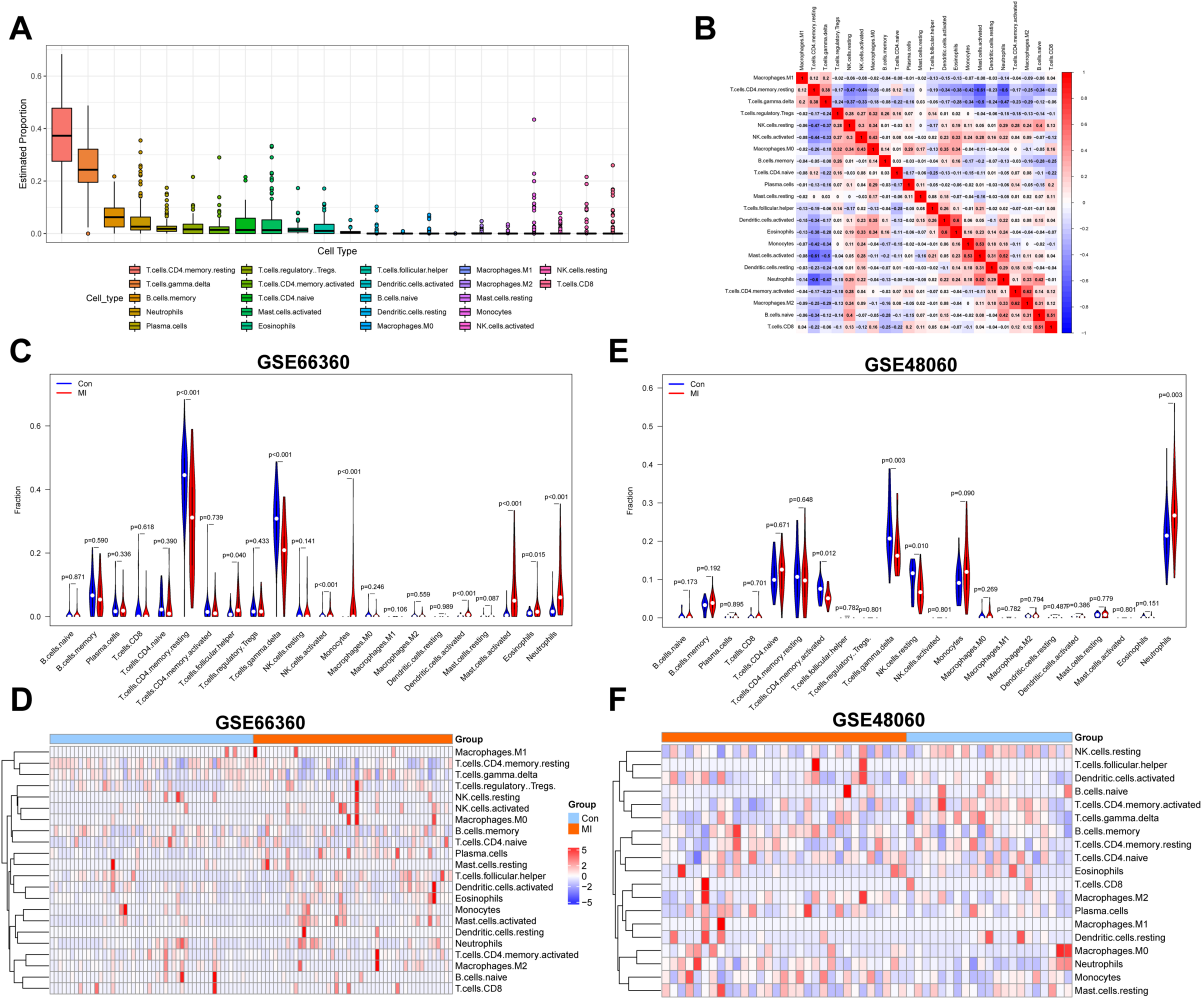
Identification of key IICs associated with MI. **(A)** A box plot created using GSE66360 data shows proportions of IICs in MI and normal tissues. **(B)** Coherence between 22 cell types present in GSE66360 data is shown in a heatmap. Red indicates high coherence, while blue indicates low coherence. **(C)** A comparison of immune cell infiltration levels between MI and normal samples from GSE66360 data is shown. Red and blue colors represent MI and normal samples, respectively. **(D)** A heatmap indicating the correlation between 22 IICs and samples within the GSE66360 dataset is shown. Red indicates a positive correlation and blue indicates a negative correlation. **(E)** A violin plot reveals proportions of 22 cell types in MI and normal tissues based on GSE48060 data. **(F)** The relevance between IICs and tissues in GSE48060. Values of *p* < 0.05 were considered statistically significant. IICs, infiltrating immune cells; MI, myocardial infarction.

The landscape of 22 IICs in MI and healthy control tissues was shown using the CIBERSORT algorithm. Proportions of IICs in each sample were determined based on GSE66360 and GSE48060 data and are shown in Supplementary Figures S1A,B, respectively. Proportions of IICs in all samples ranked by infiltration level are shown in Figure 2A; Supplementary Figure S1C. The top four IICs in GSE66360 were CD4 memory resting *T* cells, gamma delta *T* cells, memory B cells, and neutrophils, while the top four IICs in the GSE48060 dataset were neutrophils, gamma delta *T* cells, CD4 naive *T* cells, and CD4 memory resting *T* cells. A correlation heatmap of 22 types of IICs identified in the GSE66360 dataset revealed that neutrophils were negatively correlated with *T* cells and CD4 memory resting cells, and positively correlated with activated mast cells. GSE66360 dataset analysis revealed that gamma delta *T* cells were negatively correlated with *T* cells and CD4 memory resting cells, and positively correlated with activated mast cells (Figure 2B). Correlations between 19 types of IICs were determined using GSE48060 data and were shown in Supplementary Figure S1D, three IICs that had not infiltrated were excluded. Neutrophils were significantly positively correlated with m0 macrophages and negatively correlated with gamma delta *T* cells. Gamma delta *T* cells were positively correlated with *T* cells and CD4 memory activated cells, and negatively correlated with neutrophils. Differences in immune cell infiltration levels between MI and healthy control samples in the GSE66360 dataset are shown in Figure 2C. Infiltration levels of CD4 memory resting *T* cells (*p* < 0.001) and gamma delta *T* cells (*p* < 0.001) were reduced, while those of mast cells (*p* < 0.001) and neutrophils (*p* < 0.001) were increased vs. healthy control samples. We explored whether expression levels of these genes were associated with the 22 types of IICs found to be overrepresented in MI. A heatmap revealed that expression levels of genes upregulated in MI were positively correlated with IICs, including neutrophils and activated mast cells. In contrast, these genes were negatively correlated with CD4 memory resting and gamma delta *T* cells (Figure 2D). Differences between IIC levels within MI and healthy control tissues that were revealed using GSE48060 data are shown in Figure 2E. A violin plot shows that in MI samples, neutrophils (*p* = 0.003) exhibited increased levels of infiltration, while gamma delta T cells (*p* = 0.003) and resting NK cells (*p* = 0.010) infiltrated less vs. healthy control samples. Finally, a heatmap created using GSE48060 data revealed that expression levels of identified genes were associated with IICs (Figure 2F). CD4 memory-activated T and gamma delta cells were negatively correlated with gene expression levels, while neutrophils were positively correlated.

### 3.2 Identification of DEGs

GSE66360 and GSE48060 datasets were downloaded from the GEO database, both of which used the same platform. In total, 22,881 DEGs were identified when 49 MI and 50 healthy control samples were compared using GSE66360 dataset. Thereafter, 1,047 significantly upregulated and 1,396 significantly downregulated genes were identified. A volcano plot was constructed to visualize gene expression distributions (Supplementary Figure S2A). Expression levels of the 50 most highly upregulated and downregulated genes were revealed using a heat map (Supplementary Figure S2B). Genes in which |logFC| > 0.5 and *p* < 0.05 were considered statistically significant DEGs. PCA was used to compare differences in gene expression patterns of MI and control groups using GSE66360 data (Supplementary Figure S2C). The results revealed that genes from MI and healthy control samples were distributed in distinct clusters, suggesting that gene expression patterns in MI samples differed from those of healthy control samples.

### 3.3 WGCNA-based identification of key module genes

**Figure 3:**
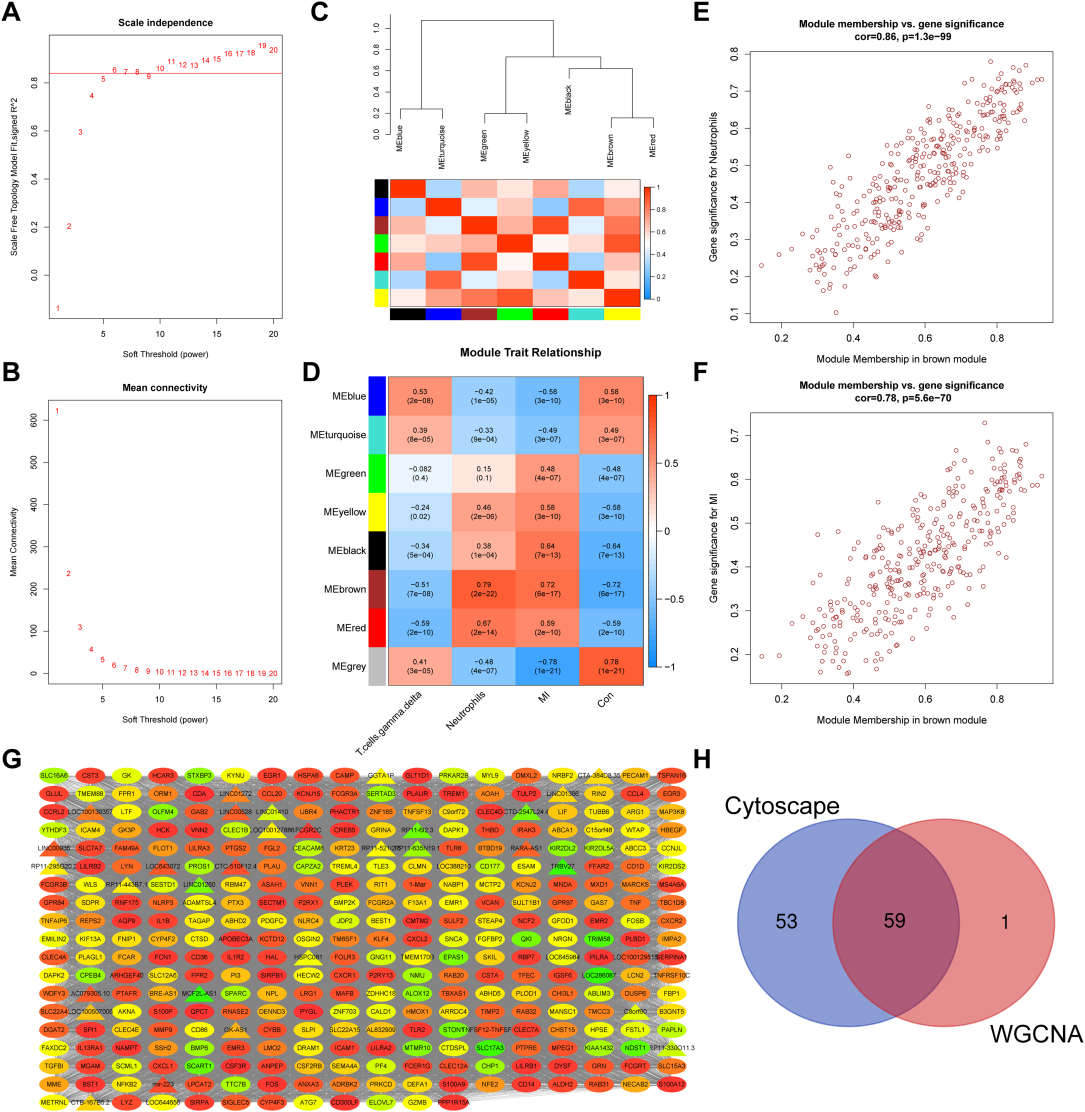
Identification of a key module via WGCNA. Scale-free index **(A)** and mean connectivity **(B)** analyses used to obtain various soft-thresholding powers are shown. **(C)** A dendrogram, heatmap of module genes, and a **(D)** heatmap showing the correlation between different modules and traits are shown. Every row stands for a module, and every column represents a trait. Spearman's correlation and *P* values are represented by the numbers in each cell. **(E**,**F)** A scatter plot analysis showing associations between GS and MM in the brown module is shown. Module genes with GS > 0.6 and MM > 0.7 values were considered key genes. **(G)** A WGCNA co-expression network of brown module genes is shown. LncRNA are indicated by triangles and mRNA by ovals. Degree of gene connectivity is indicated by the color of the nodes from low(green) to high(red). **(H)** A Venn plot revealing key genes is shown. Values of *p* < 0.05 were considered statistically significant. GS, gene significance; MM, module membership; WGCNA, weighted gene correlation network analysis.

Identified DEGs were screened to find key modules and genes associated with MI. First, hierarchical clustering analysis was conducted to remove outliers (Supplementary Figure S3A). The pickSoftThreshold function was used during WGCNA to assess the topology of the network. A soft threshold parameter was set at six, and a scale-free R2 of 0.85 was used to construct a scale-free network (Figure 3A) with high-average connectivity (Figure 3B). Relationships between identified modules were mapped. The TOM of all DEGs was displayed using a heatmap (Supplementary Figure S3B). Light colors indicate a low degree of overlap and red represents a high degree of overlap. DEGs with similar expression patterns were placed in an independent module using average linkage hierarchical clustering. As shown in a clustered dendrogram, seven modules were identified after merging dynamic modules and setting minModuleSize to 30, deepSplit to 2, and mergeCutHeight 0.15 (Supplementary Figure S3C). The gray module included non-expressed DEGs, which were excluded from further analyses. The heatmap and clustered tree indicated that expression levels of genes in each module were relatively independent of those in the other modules. We then clustered genes that could provide information about between-module relationships to analyze connectivity. The dendrogram and heatmap revealed that seven modules could be divided into two clusters (Figure 3C, Supplementary Table S1). Relatively high degree connectivity was observed between two sets of modules: brown with red modules and green with red modules. Subsequently, Spearman's correlation coefficients of the seven modules and clinical characteristics were calculated to identify the most significant associations (Figure 3D). As shown in the heatmap, brown module genes were significantly correlated with neutrophils (R = 0.79, *p* = 2e−22) and MI (R = 0.72, *p* = 6e−17). Two scatterplots of module membership vs. gene significance also showed that correlation coefficients between genes in the brown module and neutrophils (Figure 3E, co*r* = 0.86, *p* = 1.3e−99) and MI (Figure 3F; co*r* = 0.78, *p* = 5.6e−70) were high. The network of genes in the brown module is shown in Figure 3G. Sixty genes with GS > 0.6 and MM > 0.7 were identified for further analysis. Finally, we conducted the “NetworkAnalyzer” function in Cytoscape to calculate the degree of nodes in the network. A total of 112 nodes with degree >300 were selected based on their intersection with 60 genes, as is depicted in the Venn plot shown in Figure 3H.

### 3.4 Functional enrichment analysis and construction of PPI network

**Figure 4:**
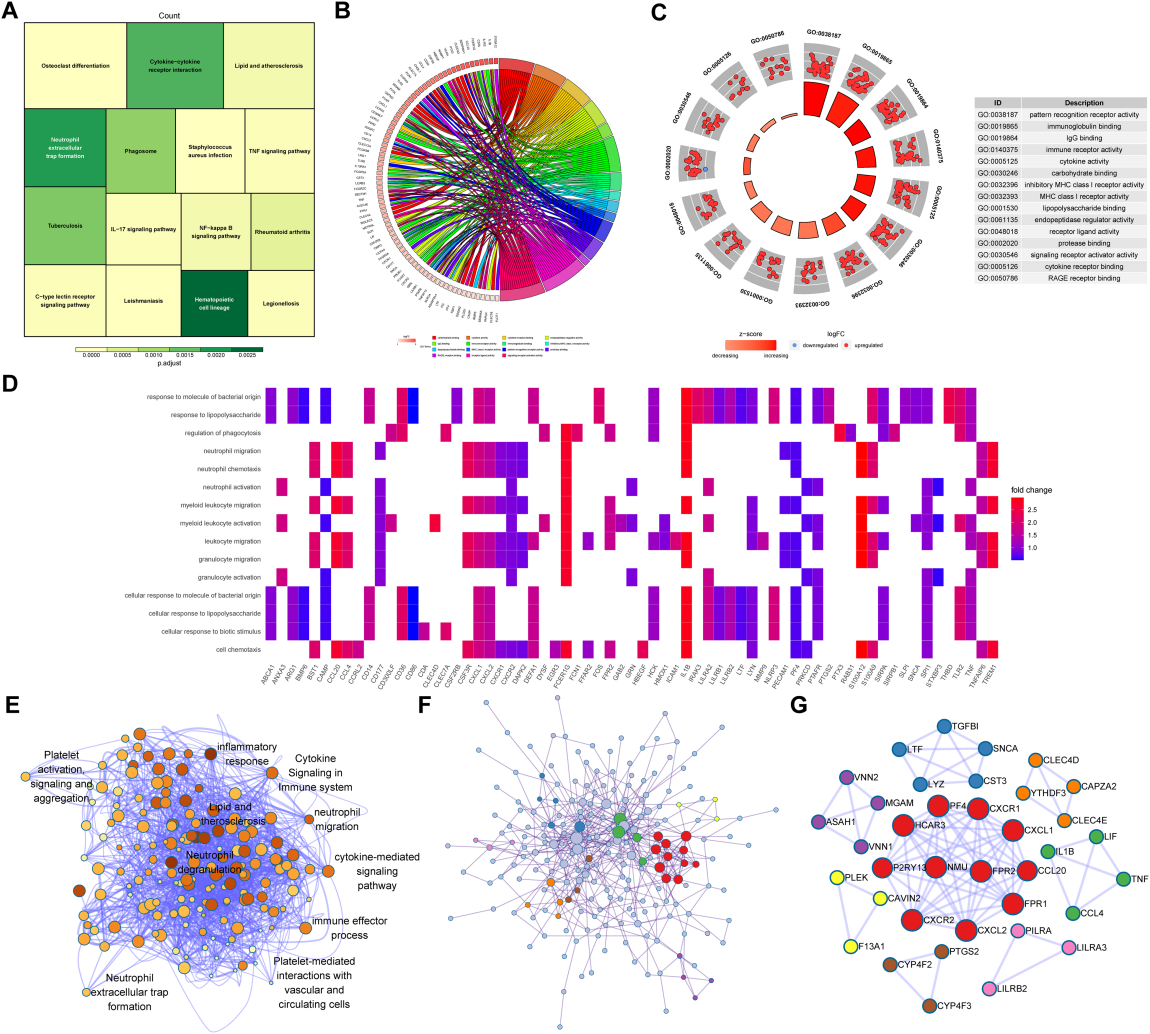
Functional enrichment analysis based on brown module genes. **(A)** A Treeplot of the top 15 most significant KEGG pathways and the top 15 GO terms identified via GO functional analysis is shown. **(B)** A chord diagram of CC, **(C)** circle graph of MF, **(D)** heatmap of BP are shown. **(E)** Underlying biological processes and related signaling pathways enriched in the brown module were identified using Metascape. **(F)** A PPI network of brown module proteins and **(G)** hub networks of the PPI network created using an MCODE plug-in are shown. KEGG, kyoto encyclopedia of genes and genomes; GO, gene ontology; BP, biological processes; CC, cell component; MF, molecular function; PPI, protein-protein interaction; MCODE, molecular complex detection.

Functional enrichment analysis was performed to explore potential biological functions and pathways of identified genes. KEGG pathway enrichment analysis indicated that identified DEGs were mainly involved in cytokine-cytokine receptor interactions, neutrophil extracellular trap formation, lipid and atherosclerosis, and the IL-17 signaling pathway, as shown in Figure 4A. GO enrichment analysis was performed from the following three perspectives: biological process (BP), cell composition (CC), and molecular function (MF). The top 15 GO terms associated with DEGs were shown in Figures 4B–D. Identified GO terms included pattern recognition receptor activity, immunoglobulin binding, IgG binding, immune receptor activity, cytokine activity, carbohydrate binding, and protease binding (Supplementary Table S2). The Metascape algorithm was used to investigate immune-related pathways, vessel-related pathways, and their interactions, including neutrophil extracellular trap formation, neutrophil migration, cytokine-mediated signaling pathways, and platelet-mediated interactions with vascular and circulating cells, lipids, and atherosclerosis (Figure 4E). Finally, an underlying regulatory network was revealed via PPI analysis (Figure 4F), with hub genes extracted from the PPI network using the MCODE plug-in of Cytoscape (Figure 4G).

### 3.5 Selection of hub genes via machine learning

**Figure 5:**
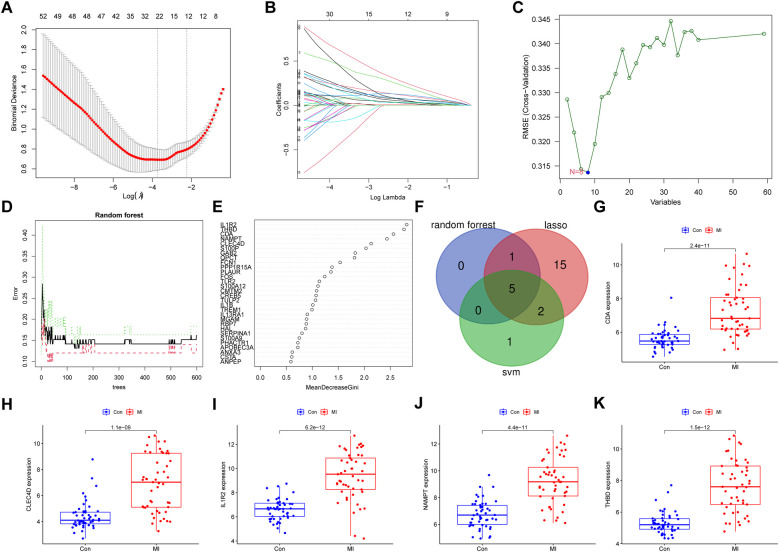
Identification of hub genes by three machine learning algorithms and verification. **(A**,**B)** LASSO regression and **(C)** SVM-RFE algorithm analysis to screen for genes involved in MI are shown. The blue dot reveals the number of genes with the smallest degree of cross-validation. **(D)** Random forest analysis performed to calculate error rate based on the number of trees and **(E)** the mean decrease Gini are shown. **(F)** Genes identified based on their identification via three distinct analyses are shown. (**G**–**K**) Expression levels of the five key genes identified via comparisons between MI and normal samples, verified using GSE66360 data, are shown. Values of *p* < 0.05 indicated statistical significance. LASSO, least absolute shrinkage and selection operator; SVM-REF, support vector machine recursive feature elimination.

After the intersection of the 112 nodes and 60 key genes, a total of 59 genes were identified. Three machine algorithms were used to identify hub genes, as follows: LASSO regression analysis, SVM-RFE, and a random forest algorithm. Twenty-three genes were selected from 59 genes using LASSO regression analysis (Figures 5A,B), eight genes were identified by SVM-REF analysis (Figure 5C), and six genes were selected using a random forest algorithm (Figures 5D,E; Supplementary Table S3). Five genes were identified by combining results of all three methods (Figure 5F): interleukin 1 receptor type II (IL1R2), C-type lectin domain family 4, member D (CLEC4D), cytidine deaminase (CDA), thrombomodulin (THBD), and nicotinamide phosphoribosyltransferase (NAMPT).

### 3.6 Validation of hub genes

Using GSE66360 and GSE48060 data, we evaluated whether the five hub genes were expressed differently in samples derived from MI tissues vs. healthy controls. As shown in Figures 5G–K, GSE66360 data revealed that expression levels of each of the five genes from MI tissues were significantly higher than those from healthy controls (*p* < 0.05). However, GSE48060 data revealed that four of the five genes were more highly expressed in MI samples than in healthy controls, while THBD was upregulated in MI samples, though not significantly (*p* > 0.05; Supplementary Figures S4A–E).

### 3.7 Diagnostic accuracy of key genes

**Figure 6:**
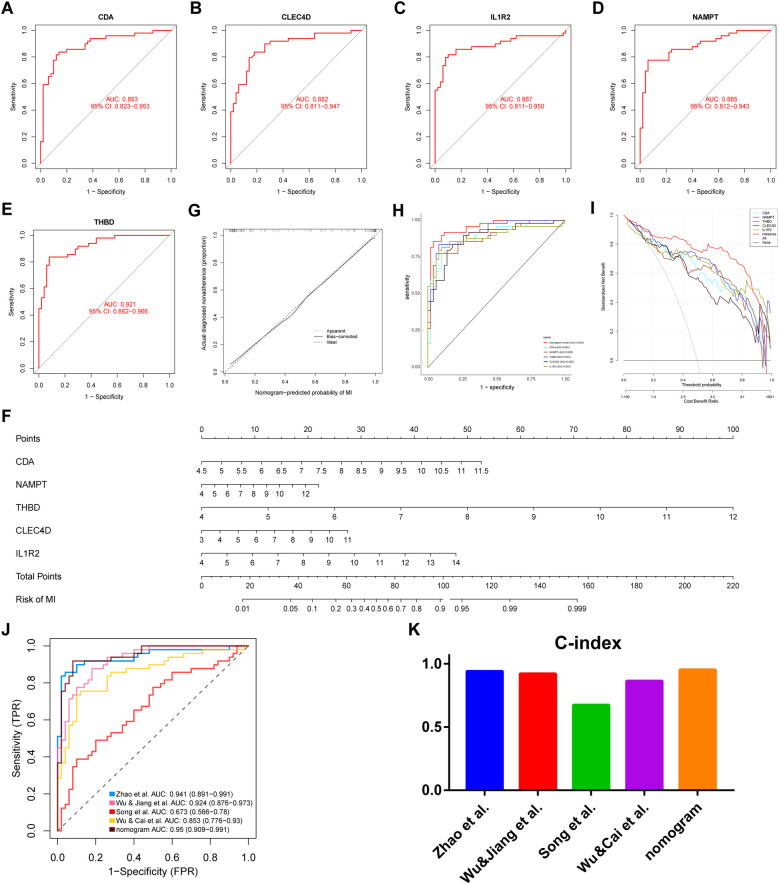
Evaluation of hub genes, construction and validation of a predictive nomogram for MI and comparison of AUC and C-index values of this model vs. those of other articles. ROC curves of CDA **(A)**, CLEC4D **(B)**, IL1R2 **(C)**, NAMPT **(D)**, and THBD **(E)** are shown. **(F)** Hub genes integration to establish a diagnostic nomogram to predict risk of MI is shown. **(G)** A calibration curve comparing predicted and actual observations, **(H)** the predictive efficiency of the nomogram and other hub genes evaluated via ROC, and **(I)** DCA curves of the nomogram and other hub genes are shown. The ‘none plot’ represents no patients were diagnosed and the ‘all plot’ represents all patients were diagnosed. **(J)** ROC curves of models from four prior studies and our study and **(K)** C-index values from models of four prior articles vs. that of our model are shown. ROC, receiver operating characteristic; DCA, decision curve analysis; AUC, area under the ROC curve.

ROC curves were drawn to estimate the predictive accuracy of the identified genes using the GSE66360 dataset. The area under the ROC curve (AUC) values for CDA, CLEC4D, IL1R2, NAMPT, and THBD were 0.893, 0.882, 0.887, 0.885, and 0.921, respectively (Figures 6A–E). These values demonstrating that each of the five genes had good predictive ability. To improve the convenience of diagnosing MI, we constructed a nomogram using the five genes, as shown in Figure 6F. C-index was used to test the predictive ability of the diagnostic model, revealing a C-index value of 0.953, a value indicating high accuracy. Subsequently, strong consistency between predicted and observed outcomes was demonstrated via the calibration curve of the predictive nomogram used to determine risk associated with MI (Figure 6G). ROC curves of all five genes and the nomogram are shown in Figure 6H. DCA curves of key genes and the nomogram were drawn to determine whether the key genes could improve clinical decision-making regarding the diagnosis of MI (Figure 6I). Furthermore, to verify the results, we used the five key genes to build a nomogram to predict a risk score for MI based on GSE48060 data (Supplementary Figure S5A) and a calibration curve was constructed to assess the accuracy of the predictive model (Supplementary Figure S5B). The diagnostic accuracy of the nomogram model was better than that of conventional methods. The AUC of the nomogram model was 0.953, indicating an excellent predictive ability for MI. A ROC curve drawn using calculated AUC values of key genes identified in four other articles revealed that the AUC of our model was higher than those of other studies (Figure 6J). Finally, C-index values of the key genes identified in the studies were calculated and were shown in Figure 6K. We discovered that the C-index value of our model was higher than those of other studies.

### 3.8 GSEA

GSEA based on GSE66360 data was used to identify molecular mechanisms potentially involved in MI. Results revealed that in patients at high risk of MI, pathways related to the chemokine signaling pathway, B cell receptor signaling pathway, cytokine receptor interaction, FC gamma R-mediated phagocytosis, leukocyte transendothelial migration, and natural killer cell-mediated cytotoxicity were enriched (Supplementary Figures S6A–F). Supplementary Figure S6G showed pathways enriched in patients with low-risk MI. These pathways included nucleotide excision repair, oxidative phosphorylation, proteasomes, ribosomes, spliceosomes, and ubiquitin-mediated proteolysis.

### 3.9 Comparison of hub gene and IIC risk scores

**Figure 7:**
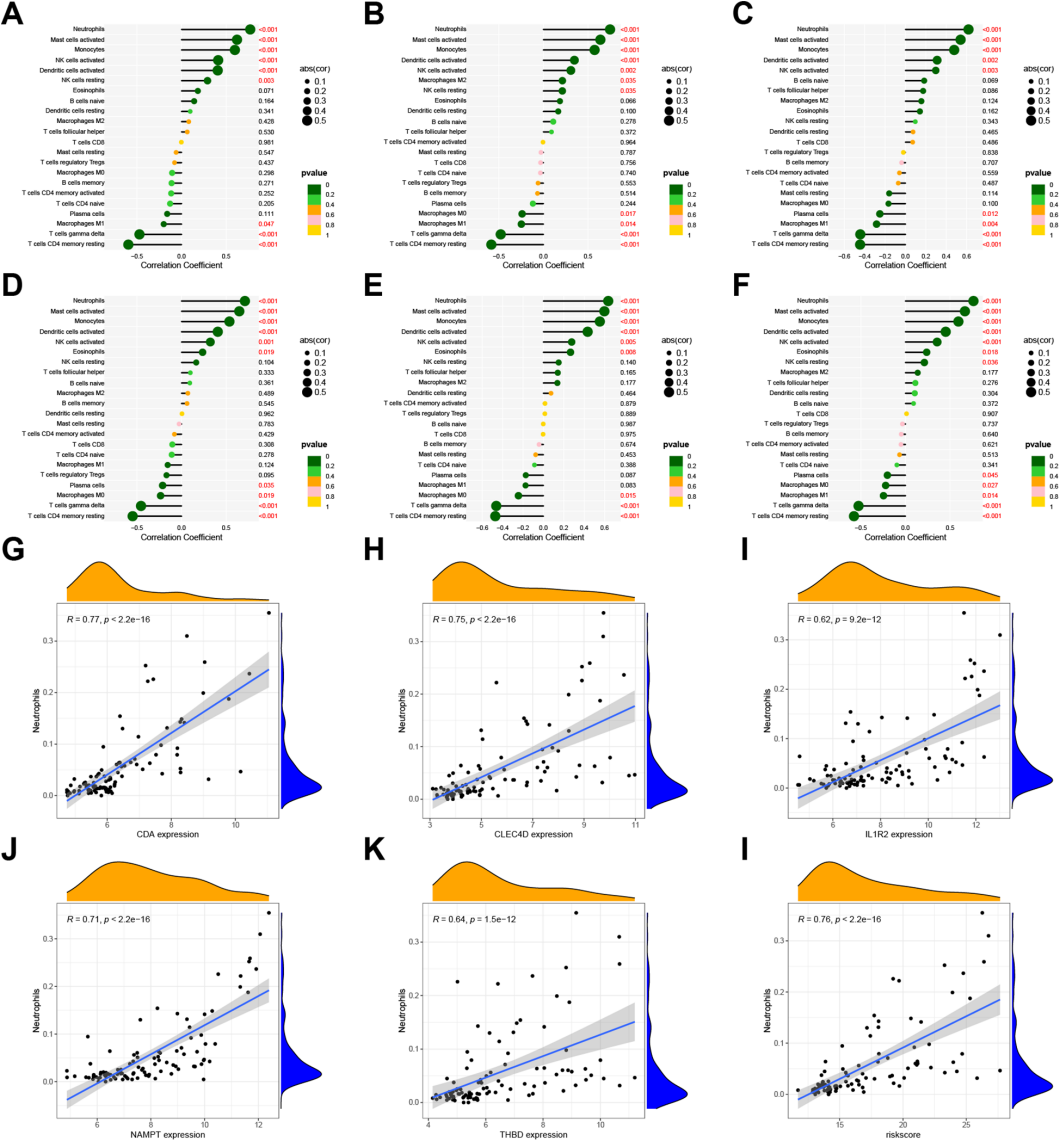
Correlation between 22 IICs and expression levels of hub genes using GSE66360 data. Lollipop plots indicating the relationship between IICs and CDA **(A)**, CLEC4D **(B)**, IL1R2 **(C)**, NAMPT **(D)**, THBD **(E)**, and risk score for MI **(F)** are shown. Scatter plots revealing the correlation coefficient between neutrophils and expression levels of CDA **(G)**, CLEC4D **(H)**, IL1R2 **(I)**, NAMPT **(J)**, THBD **(K)**, risk score of MI **(L)**. Values of *p* < 0.05 were considered statistically significant. IIC, infiltrating immune cell; MI, myocardial infarction.

The regression coefficients of each gene were calculated using logistic regression. Spearman correlation analysis-based relationships between key genes and IICs were displayed in Figures 7A–E. CDA was significantly and positively correlated with neutrophils, activated mast cells, activated monocytes, activated NK cells, and activated DCs (*p* < 0.05; Figure 7A). CLEC4D expression was significantly and positively correlated with activated neutrophils, mast cells, monocytes, and DCs (Figure 7B). IL1R2 expression was positively correlated with neutrophils, activated mast cells, and monocytes (Figure 7C). NAMPT expression showed a significant, positive correlation with neutrophils, activated mast cells, monocytes, and DCs (Figure 7D). THBD expression was positively correlated with activated neutrophils, mast cells, monocytes, and DCs (Figure 7E). Expression of the five identified genes was negatively correlated with gamma delta *T* cells and CD4+ memory resting *T* cells. Nomogram risk scores revealed a significant, positive relationship with neutrophils, activated mast cells, monocytes, activated DCs, and activated NK cells, and a significant, negative correlation with gamma delta *T* cells and CD4 memory resting *T* cells (Figure 7F, Supplementary Table S4). As shown in Figures 7G–L, CDA, CLEC4D, IL1R2, NAMPT, THBD, and the nomogram were significantly positively correlated with neutrophils (*p* < 0.05 for all, R = 0.77, 0.75, 0.62, 0.71, 0.64, and 0.76, respectively).

### 3.10 Consensus clustering analysis and gene set variation analysis

**Figure 8:**
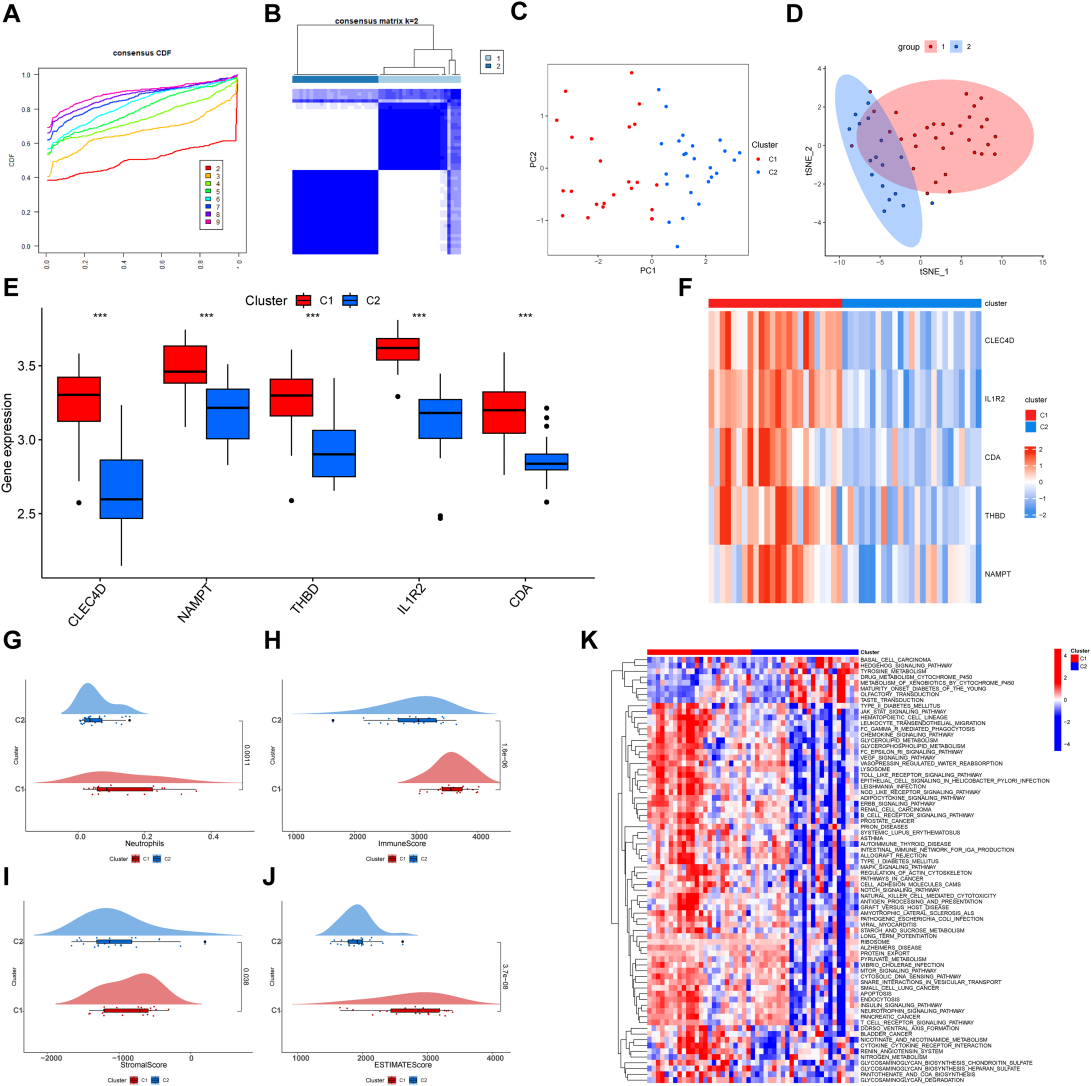
Consensus clustering analysis of genes identified from MI samples using GSE66360 data. **(A)** A CDF plot shows cumulative distribution functions for differing values of k. The plot is used to identify the optimal value of k that improves the reliability of the clustering analysis by identifying the point at which the CDF reaches its approximate maximum. **(B)** A heatmap of the consensus matrix reveals two clusters of patients. The “ConsensusClusterPlus” package was used for clustering. Colors indicate the two MI subtypes. **(C)** A PCA of the two MI subtypes is shown. Red dots indicate cluster 1 and blue dots indicate cluster 2. **(D)** The t-SNE of two MI clusters is shown. **(E)** Differing expression levels and a **(F)** heatmap displaying expression level differences of key genes in each cluster are shown. Upregulated genes are shown in red and downregulated genes are shown in blue. **(G)** A violin plot revealing the percentage of infiltrating neutrophils in each of the two clusters is shown. Violin plots of **(H)** immune, **(I)** stromal, and **(J)** estimate scores of the two clusters are shown. **(K)** Heatmap shown the signaling pathways enriched between two subtypes. MI, myocardial infarction. CDF, cumulative distribution function; PCA, principal component analysis; t-SNE, t-Distributed stochastic neighbor embedding.

Unsupervised clustering analysis was performed using data from 49 MI tissues samples in the GSE66360 dataset to classify patients into different clusters. A k value of two was selected after estimating a consensus heatmap (Figure 8B), with relative changes ranked according to the cumulative distribution function (CDF). The CDF reached an approximate maximum of k = 2, and the cluster analysis was more reliable (Figure 8A). We identified two distinct MI patterns and thereby divided patients into two corresponding clusters, as follows: 24 cases in MI-related cluster 1 and 25 cases in MI-related cluster 2 (Supplementary Table S5). PCA and t-SNE analyses were used to validate the independent distribution of MI-related subtypes (Figures 8C,D). The boxplot and heatmap revealed that the expression levels of the five hub genes were higher in cluster one than those in cluster two (Figures 8E,F). A violin plot showed that the infiltration fraction of neutrophils was higher in cluster one than that in cluster two (Figure 8G). Subsequently, the “PupillometryR” package was applied to calculate immune, stromal, and estimate scores of subtypes. As violin plots showed, immune (Figure 8H), stromal (Figure 8I), and estimated (Figure 8J) scores of cluster one were high and those of cluster two were low. Finally, we used the GSVA to evaluate the differences of biological functions in two clusters. The heatmap revealed that pathways related to immune and inflammations, such as leukocyte transendothelial migration, B cell receptor signaling pathway, T cell receptor signaling pathway were enriched in cluster 1, while metabolism pathways were enriched in cluster 2 (Figure 8K). Furthermore, to access the stability of subtypes, the GSE48060 dataset was used for validation. The result displayed that the clusters were independent of each other (Supplementary Figures S7A–C). Meanwhile, we found the key genes and neutrophils were significantly distributed between the two subtypes, demonstrating a high level of immune heterogeneity (Supplementary Figures S7D–F).

### 3.11 Identification of MiRNAs and construction of CeRNA network in MI

To identify miRNAs that may bind to the five key genes, miRNAs appearing in more than one database were considered (Supplementary Figures S8A–E). Results showed that 230 miRNAs bind hub genes. A ceRNA network was constructed to display potential interactions between miRNAs and key genes (Supplementary Figure S8F).

### 3.12 Single-cell RNA sequencing analysis

**Figure 9:**
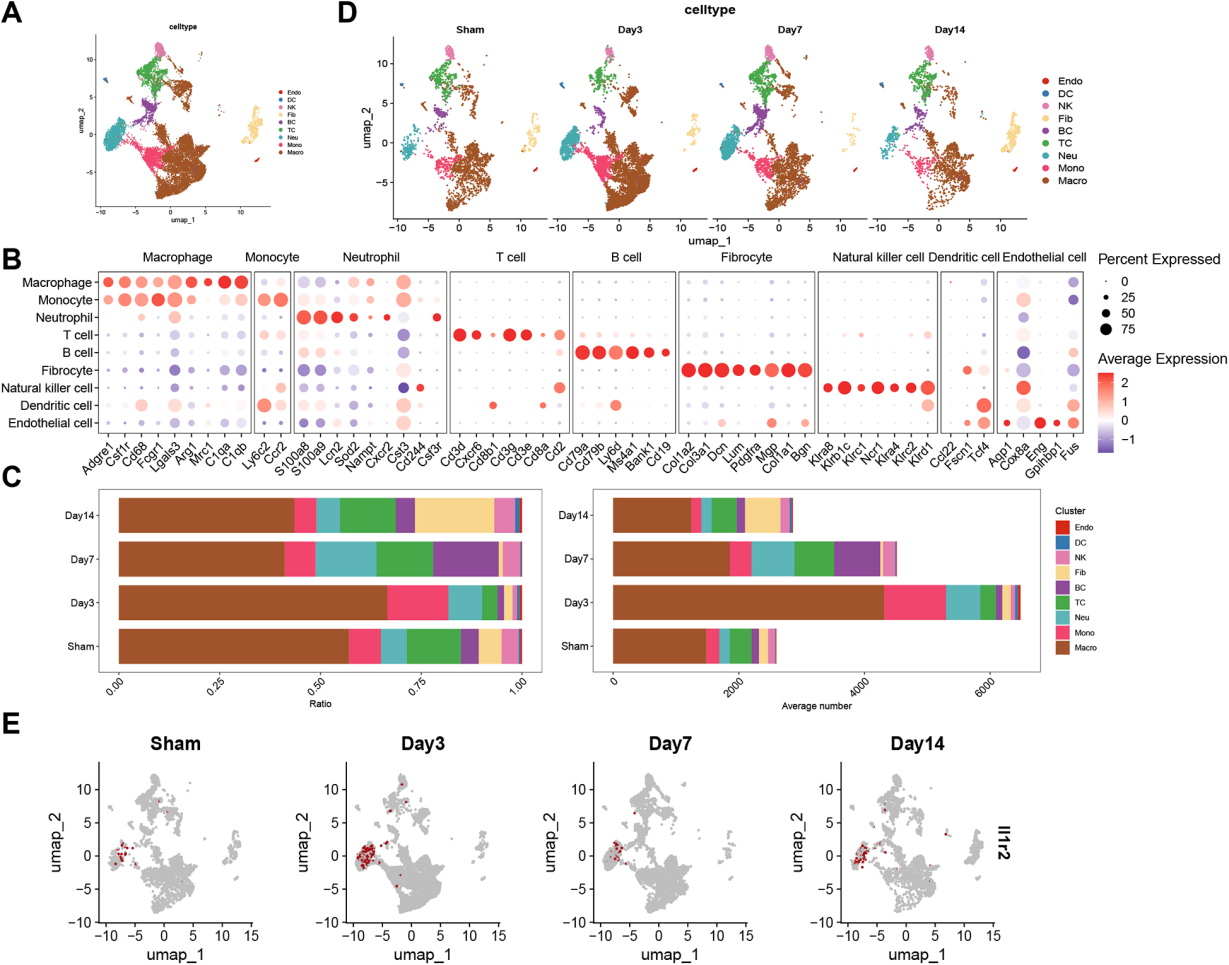
Single-cell RNA sequence analysis. **(A)** UMAP visualization of 9 cell types in control and infarcted heart tissue from mice. **(B)** Dot plot shown the expression of markers of each cell type. The percentage that every cell cluster expressed the marker genes was represented with dot size and the expression level was represented with dot color. **(C)** Stack plots revealed the ratio and absolute number of different cell types in sham, 3 days, 7 days and 14 days after myocardial infarction groups. UMAP plots visualized the genes of each cell type **(D)** and the expression of IL1R2 **(E)** in sham, day3, day 7 and day14 post-MI groups. UMAP, Uniform manifold approximation and projection; Sham, sham-operated.

Cluster analysis divided scRNA-seq cells into 14 clusters (Supplementary Figure S9B). The 14 clusters were categorized into 9 types of cells by markers (Figure 9A; Supplementary Figure S9A). The Figure 9B revealed the expression of markers in each cell. Then, the ratio and the absolute number of cells in samples were displayed in Figure 9C. The stack plot showed the ratio of neutrophils was higher on the 7th day after MI. Interestingly, on the 14th day after MI, the ratio of neutrophils decreased. As the UMAP plot displayed, the expression of genes of each cell was calculated in sham, day3, day7 and day14 post-MI groups (Figure 9D). We found the genes of neutrophil increased on the seventh day, and decreased on the fourteenth day after MI. Subsequently, as the Supplementary Figure S9C revealed, the expression of IL1R2 was higher in neutrophil than in other cells and more concentrated in neutrophil compared to other hub genes. The expression of IL1R2 increased on the 3rd day and decreased on the 7th day after MI (Figure 9E). Finally, the ratio of IL1R2 in each group was calculated, Sham: 0.087, D3: 0.228, D7: 0.111, D14: 0.195.

### 3.13 Immunofluorescence and immunohistochemistry

**Figure 10:**
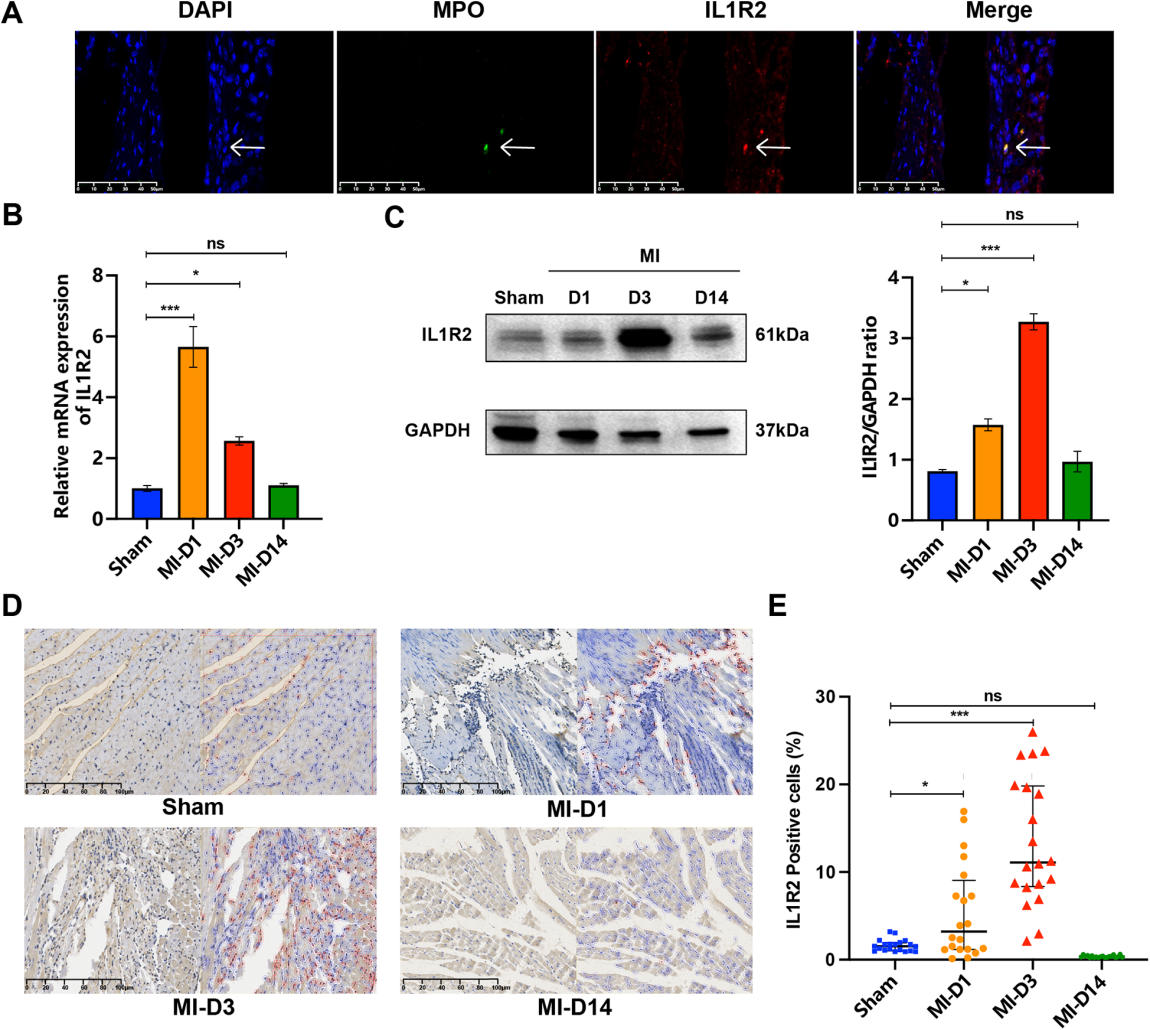
Detection of IL1R2 in control and infarcted murine heart tissue. **(A)** IF staining for IL1R2 (red) and MPO (green) with DAPI (4’,6-diamidino-2-phenylindole) (blue) nuclear counterstain in normal and infarcted tissue. **(B)** The mRNA expression of IL1R2 from sham group and MI group was detected by qPCR and normalized to GAPDH expression values were expressed as mean ± standard deviation (mean ± SD). **(C)** The protein expression level of IL1R2 in the mice heart from sham group and MI groups were detected by WB. **(D)** IHC plot of IL1R2 positive cells in sham, D1, D3, D14 tissue. The positive cells were represented by red circles. **(E)** The scatter plot revealed the difference of IL1R2 positive cells in sham, D1, D3, D14 heart tissue. *p < 0.05; **p < 0.01; ***p < 0.001. IF, immunofluorescence; IHC, immunohistochemistry.

We examined the distribution of Il1r2 in sham-operated group and different stages of myocardial infarction, 1 day, 3 days, 14 days after MI, and found Il1r2 was co-expressed with MPO, as the fluorescent microscopy analysis displayed (Figure 10A). PCR analysis revealed that the expression of IL1R2 was significantly higher in MI tissues compared to normal tissues. IL1R2 expression peaked on the first day following MI and subsequently decreased over time (Figure 10B). WB results showed that, compared to the sham group, the expression of Il1r2 was higher on both the first and third days after MI. Additionally, the expression of Il1r2 on the third day post-MI was higher than on the first day. Two weeks after MI, the expression of Il1r2 was lower than on both the first and third days post-MI (Figure 10C). Then, as the IHC staining showed, compared to sham group, the positive rate of Il1r2 was higher on the first day (*p* < 0.05) and third day after MI (*p* < 0.001), demonstrating the diagnosis value of Il1r2. Moreover, the rate of Il1r2 was also higher on the third day after myocardial infarction than the first day, which revealed the potential of Il1r2 in predicting the progression of MI. Interestingly, two weeks after MI, the Il1r2 expression level was lower than those on the first day and third day after MI (Figures 10D,E).

## 4 Discussion

Although the development of therapies has led to breakthroughs in MI treatment, the disease remains a leading cause of death worldwide (17, 18). Currently, methods used to diagnose MI are primarily based on serum biomarkers of myocardial damage. However, these biomarkers cannot provide sufficient warning of myocardial perfusion abnormalities in the early stages of MI (19). Further, myocardial injury due to other cardiomyopathies such as hypertrophic and Takotsubo cardiomyopathies can interfere with the diagnosis of MI (20, 21). In recent years, numerous biomarkers related to immune have been identified for MI diagnosis and risk assessment, such as S100 protein and Galectin-3 (Gal-3) (22). Moreover, point-of-care (POC) testing of platelet was proved to improve the diagnosis of AMI (23). Although some studies have confirmed that IICs are involved in the development of many diseases including Alzheimer's and pediatric acute myocarditis (24, 25), the role of IICs in MI remains unclear. Since we found that neutrophils play a key role in the occurrence of MI, we were able to build a diagnostic model for MI using machine learning algorithms. Through scRNA-seq analysis, we can identify a gene with more specificity of neutrophil than other genes of the model. Traditional types of MI including non-ST-elevation MI (NSTEMI) and ST-elevation MI (STEMI) fail to sufficiently classify patient risk associated with disease, and the main diagnostic approach involving percutaneous coronary intervention is invasive (17). Therefore, we defined two novel subtypes of MI with independent immune heterogeneity that may facilitate early intervention and the individualized treatment of MI.

Here, we describe a noninvasive MI screening approach that uses machine learning algorithms to detect changes in neutrophils of blood samples. To investigate the immune microenvironment of MI, CIBERSORT and scRNA-seq analysis were used to reveal infiltrating fractions of IICs in MI and non-diseased samples. We found that neutrophils were significantly up-regulated in MI samples in both training and validation sets, indicating that neutrophils may play a significant role in the onset of MI. Neutrophils, myeloid leukocyte cells accounting for 50%–70% of all circulating leukocytes in humans, are a dominant arm of the innate immune system that defends against pathogens. In chronic obstructive pulmonary disease, neutrophilic inflammation is a notable characteristic (26). In cardiovascular diseases (CVD), neutrophils can induce macrophage transformation to another phenotype, promoting angiogenesis. The process can facilitate the generation of new blood vessels in the ischemic heart to repair damage (12). This pathophysiological process is centered around neutrophil-driven repair mechanisms. Neutrophils contribute to the inflammatory response in CVDs through mechanisms such as degranulation and the release of neutrophil extracellular traps (NETs). NETs interact with endothelial cells and platelets, promoting immune thrombosis, and are implicated in the progression of various CVDs. NETs represent a promising therapeutic target for anti-inflammatory strategies in CVD (27). Neutrophils and platelets can be simultaneously activated in various cardiovascular diseases, and their interaction may serve as a potential target for novel therapeutic strategies in cardiovascular diseases (28). A recent study found that inflammatory reactions and microcirculatory disturbances associated with neutrophils that protect the heart from ischemia-reperfusion injury are mediated by PDE4B (29). Interestingly, scRNA-seq analysis revealed that the neutrophil ratio in all cells initially increased and then decreased, as shown by Jin. This is an important cellular mechanism to alleviate the inflammation in ischemic heart tissue. Meanwhile, the mechanism can activate the programs of anti-inflammation to promote the transition from ischemia to reparative stage (30). In acute coronary syndrome (ACS) patients, neutrophil count is regarded as an independent predictor of disease progression on admission (31). The number of neutrophils which is in circulation is associated with infarct size (32). The identification of changes in neutrophils in the peripheral blood is a potentially useful therapeutic strategy.

Therefore, a module sensitive to neutrophils was screened via WGCNA to explore the regulatory molecules that mediate MI. GO and KEGG pathway enrichment analyses indicated that neutrophil- and MI-related pathways such as neutrophil migration, neutrophil extracellular trap, lipid and atherosclerosis and NF-kappa B signaling were enriched in the brown module. Atherosclerosis is a disease of the arteries that can lead to MI (33). Neutrophil activation and degranulation can lead to plaque erosion, contributing to MI (34). The NF-kappa B family plays a crucial role in the process of inflammation by promoting the expression of pro-inflammatory factors (35). NF-kappa B is also considered a destabilizer of plaques (36). Machine learning methods can be applied using general learning algorithms to predict complex, large, and hard-to-tackle health problems (37). The application of machine learning algorithms can improve the accuracy of disease susceptibility and outcome prediction methods, both of which have improved significantly in recent years (38). Key genes related to MI were identified using three machine learning algorithms (LASSO regression analysis, SVM-RFE, and the random forest method) and integrated into a nomogram model to calculate and visualize risk associated with MI occurrence via a relatively simple output. The diagnostic accuracy of the nomogram model was demonstrated to be better than that of conventional methods (39). Subsequently, DCA and the calibration curve validated the stability of our nomogram model. More importantly, the AUC and C-index of the nomogram model were higher than those of the other four articles (40–43), indicating an excellent predictive ability for MI.

Cluster analysis was used to divide the patients with MI into two clusters. Subtype heterogeneity was observed when either the infiltrating fraction of neutrophils or the expression levels of five identified hub genes were considered. Notably, expression levels of the five identified MI-related genes and neutrophil infiltration percentage were elevated in cluster one vs. cluster two. In addition, both immune and stromal scores of cluster one were higher than those of cluster two. Also, the stability of the immune subtypes was validated in a dataset. The identified subtypes may allow for individualized MI therapy. Nonetheless, molecular mechanisms underlying neutrophil-related changes and risk models require further investigation.

As a high-throughput technology, scRNA-seq analysis can quantify gene expression profile of particular cell group at the level of single cell by RNA sequencing (44, 45). The analysis can describe the specific gene expression pattern of the single cell from tissues to reveal the cellular heterogeneity of the tissue (46). Through scRNA-seq analysis, we found IL1R2 exhibited greater specificity of neutrophil than other genes in the model. IL1R2 is a member of the IL-1 receptor family and is associated with immunity and inflammation (47). Some studies have shown that IL1R2 plays a role in the progression, metastasis, and poor prognosis of tumors (48, 49). In patient with coronary atherosclerosis, the injury/inflammatory damage was prevented by IL1R2 which mediated by miR-383-3p to inhibit the inflammasome signaling pathway to active in endothelial cells of coronary artery (50). In patients with STEMI, IL1R2 is associated with left ventricular remodeling (51). IL1R2 may be involved in the immune and inflammatory responses associated with coronary artery disease (CAD). Because it significantly overexpressed in peripheral blood mononuclear cells of patients with CAD and its expression is positively correlated with the SYNTAX score and oxidized low-density lipoprotein (52). In the study, IL1R2 has been shown to inhibit cardiomyocyte apoptosis during myocardial ischemia-reperfusion injury, suggesting its potential as a therapeutic target for the prevention and treatment of myocardial infarction (53).

Mouse model of MI was constructed to best investigate the pathophysiology of ischemia heart disease. The paraffin sections of MI tissue in different periods were selected to perform following experiments. The protein targets were detected and visualized with IF on each slide of section (54). IHC was an important auxiliary tool for direct diagnosis and identification of the cell linage (55). Here, as the IF shown, Il1r2 was co-expressed with MPO. The WB analysis showed that, compared to the sham group, the expression of Il1r2 was higher on both the first and third days after MI. Additionally, the expression of Il1r2 on the third day post-MI was higher than on the first day. Two weeks after MI, the expression of Il1r2 was lower than on both the first and third days post-MI. The progression of MI was revealed by IHC. With the early progression of MI, more and more myocardial cells were injured and the rate of IL1R2 increased. The results validated that IL1R2 is specific to neutrophils and might predict early progression of MI, which could serve as a potential therapeutic target. The PCR analysis revealed a reduction in the level of IL1R2 mRNA on the third day post-MI. A decrease in mRNA levels does not necessarily result in a corresponding reduction in protein expression. This may be attributed to the cell compensating for the reduction in mRNA by modulating translation efficiency, thereby maintaining or even enhancing protein synthesis. However, there are some limitations in the study. Firstly, our datasets and samples are limited, which can cause deviation in the accuracy of biomarkers. Secondly, our experiments are based on animals, not humans, which only tentatively explain IL1R2 has potential value of diagnosis and prediction of early progression of MI. The results in this study should be verified by *in vitro* experiments in future studies.

## 5 Conclusions

In conclusion, we found and verified that neutrophils are key IICs that play a crucial role in the onset of MI. The identification of IL1R2 related to neutrophils facilitates the diagnosis of MI and prediction of the early progression of MI. Furthermore, two distinct subtypes with immune heterogeneity were identified. Immune infiltration subtype classification could facilitate the development of individualized MI therapies.

## Data Availability

The original contributions presented in the study are publicly available. These data can be found here: https://www.ncbi.nlm.nih.gov/geo/, accession numbers: GSE48060, GSE66360 and GSE163465.
