# Supplementary material for: Neutrophil-related IL1R2 gene predicts the occurrence and early progression of myocardial infarction
Source: Front Cardiovasc Med. 2025 Mar 31;12:1516043. doi: 10.3389/fcvm.2025.1516043 (PMC11994735; doi:10.3389/fcvm.2025.1516043)
Supplement: Supplementary file 10 [file Datasheet5.docx]

Supplementary Material

A Novel Machine Learning, Neutrophil-Based Diagnostic Model

Classifies Immune Subtypes in Myocardial Infarction

Jieqiong Tang^1^, Xierenayi Tudi^2^, Jingbo Zhu^3^, Tianxiang Zhang^4*^ and Tongtong Shen^1*^

*** Correspondence:**Tongtong Shen, 13955016856@163.com; Tianxiang Zhang, little_gear@sjtu.edu.cn

# Supplementary Figures

##
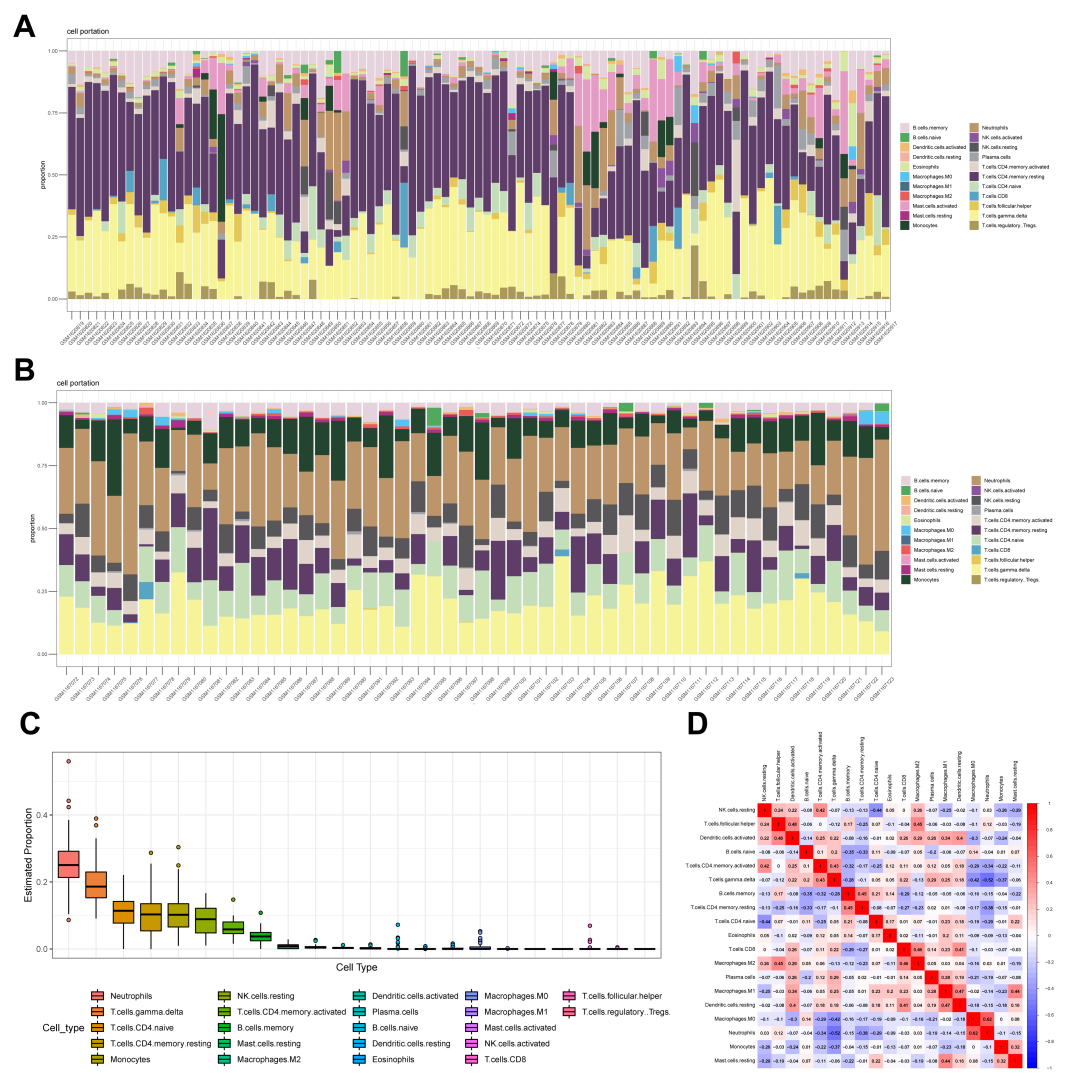


**Figure S1**: Distribution of IICs. (**A**) A stacked bar plot depicting percentages 22 cell types in every GSE66360 database sample is shown. (**B**) The percentage of IICs present in each sample included in GSE48060 data is indicated using stacked bar plot. (**C**) Proportions of 22 cell types in all samples included in GSE48060 data are shown using box plots. (**D**) A correlation heatmap displaying associations between IICs in GSE48060 data is shown. IIC, infiltrating immune cell.


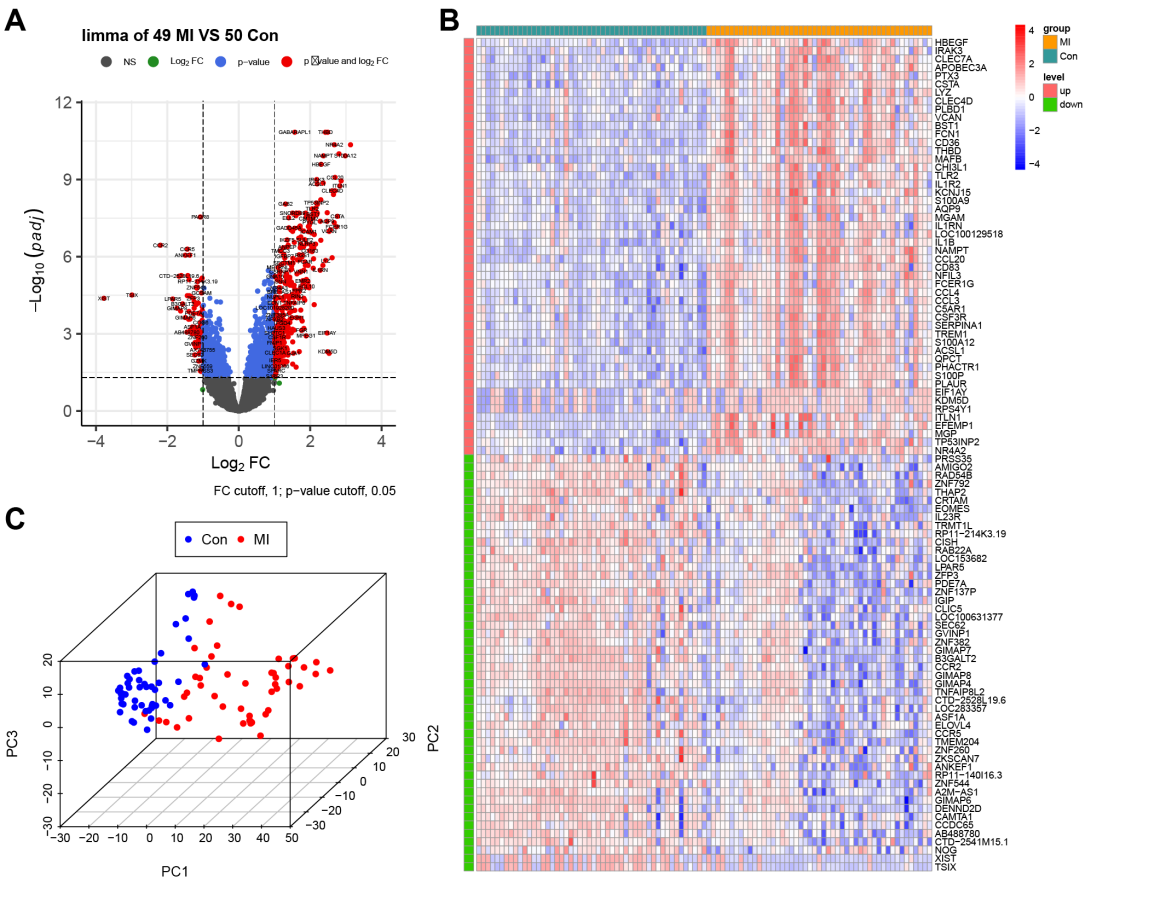


**Figure S2**. Identification of DEGs. (**A**) A volcano plot created using GSE66360 data shows DEGs detected when MI and normal tissues were compared. DEGs are displayed using red plots. Genes in which logFC > 1 are considered upregulated, while logFC values < -1 indicates downregulated genes. (**B**) A heatmap is shown that includes 100 of the most highly DEGs identified when 49 MI and 50 normal samples included in the GSE66360 dataset were compared. Upregulated genes are shown in red and downregulated genes are shown in blue. (**C**) A PCA of 49 MI and 50 normal tissues based on GSE66360 data is shown. Red dots represent MI samples and blue dots represent normal samples. DEG, differentialy expressed genes; PCA, principal component analysis; MI, myocardial infarction.


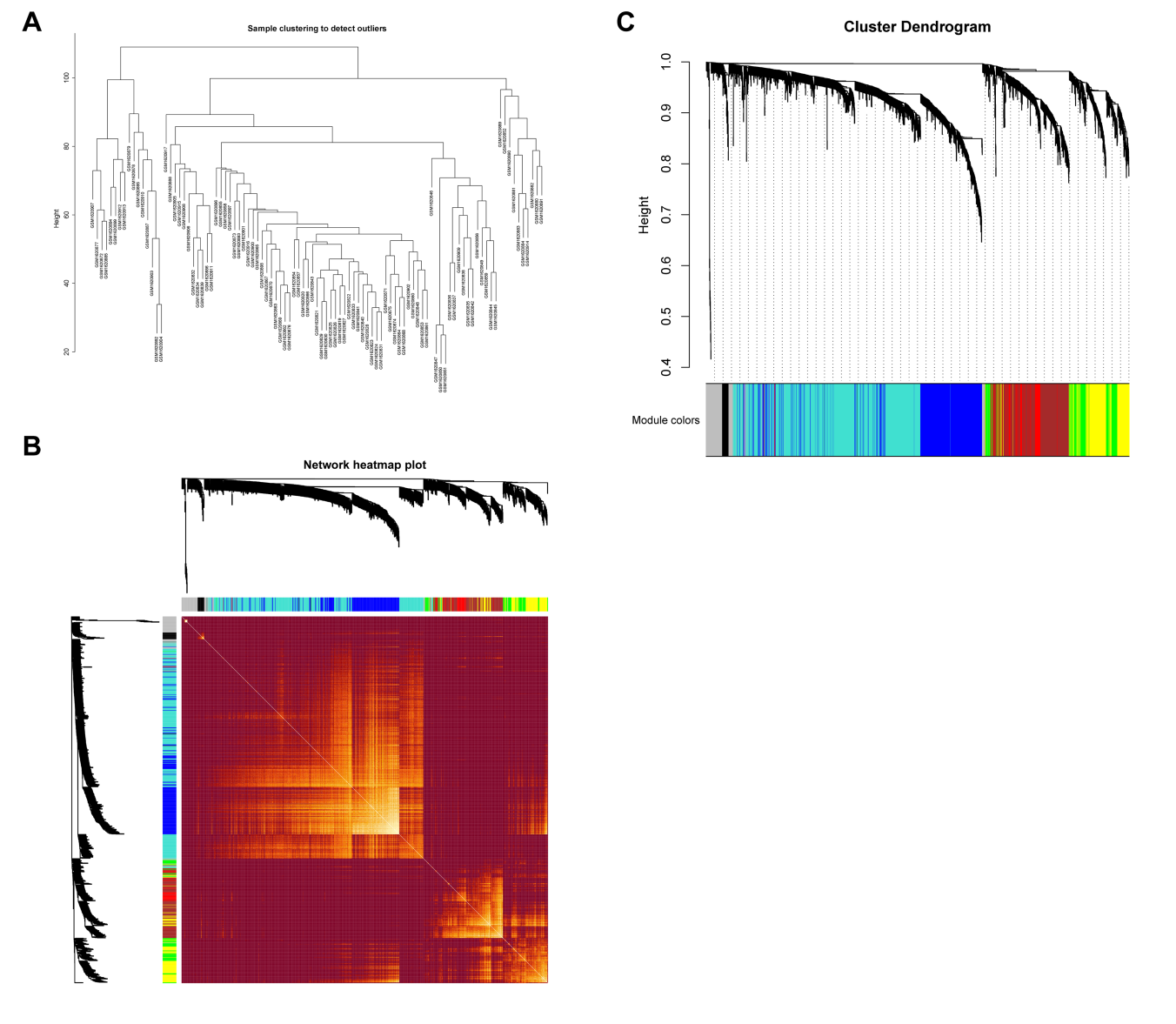


**Figure S3**: WGCNA.(**A**) Sample clustering applied for detecting outliers among 99 samples and a (**B**) cluster dendrogram of 99 tissues using measurement of dissimilarity (I-TOM), where DEGs are divided into 7 modules, and a (**C**) heatmap displaying the TOM of all modules in the WGCNA are shown. The diminishing overlap is indicated using increasingly light color and increased overlap is indicated by an increasingly bright red color. TOM, topological overlap matrix; DEG, differentialy expressed genes.


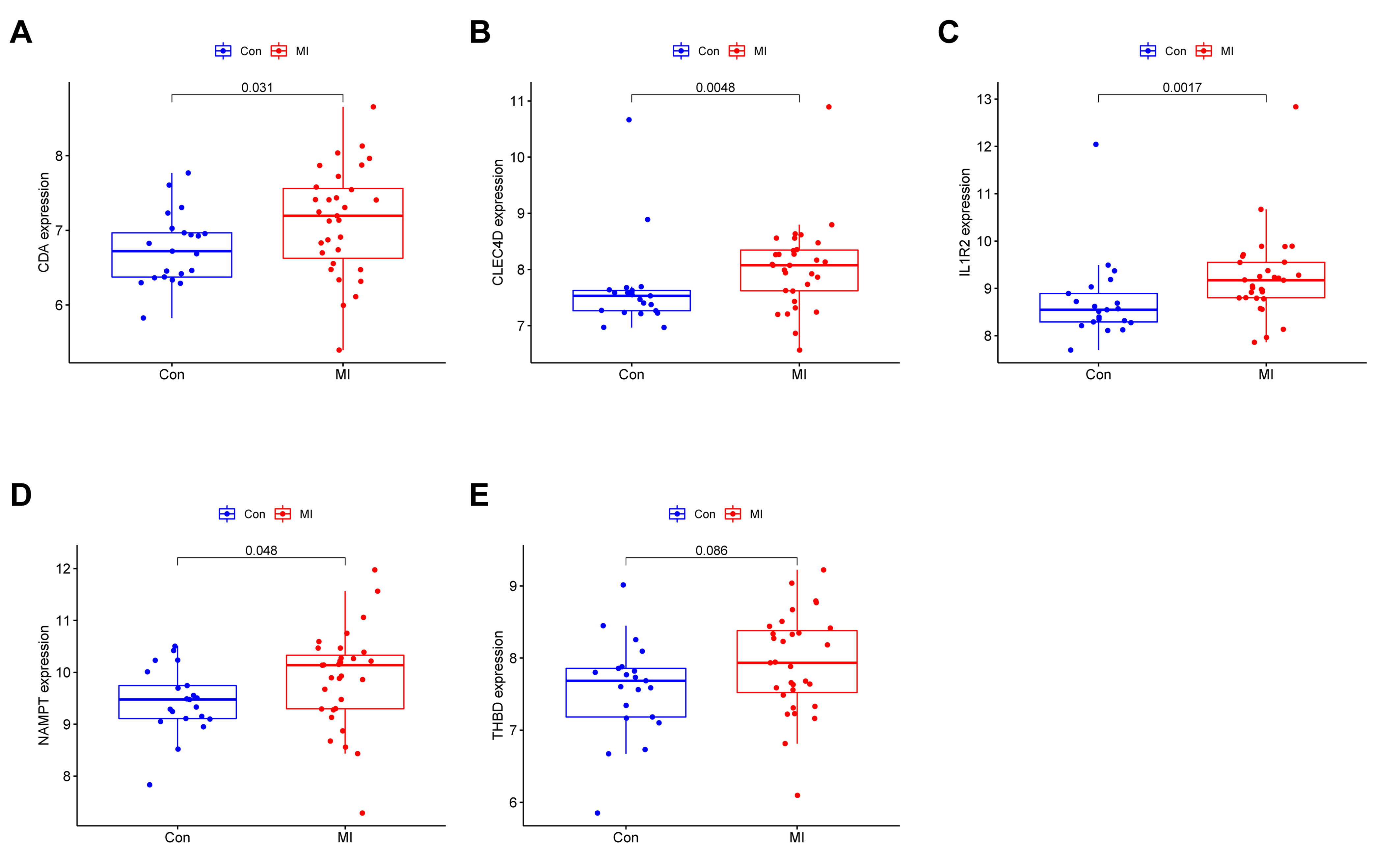


**Figure S4**: Validation of differential expression patterns of five key genes in MI and normal samples using GSE48060. Validation of CDA (**A**), CLEC4D (**B**), IL1R2 (**C**), NAMPT (**D**), THBD (**E**) expression patterns are shown. Values of p < 0.05 indicate statistical significance.


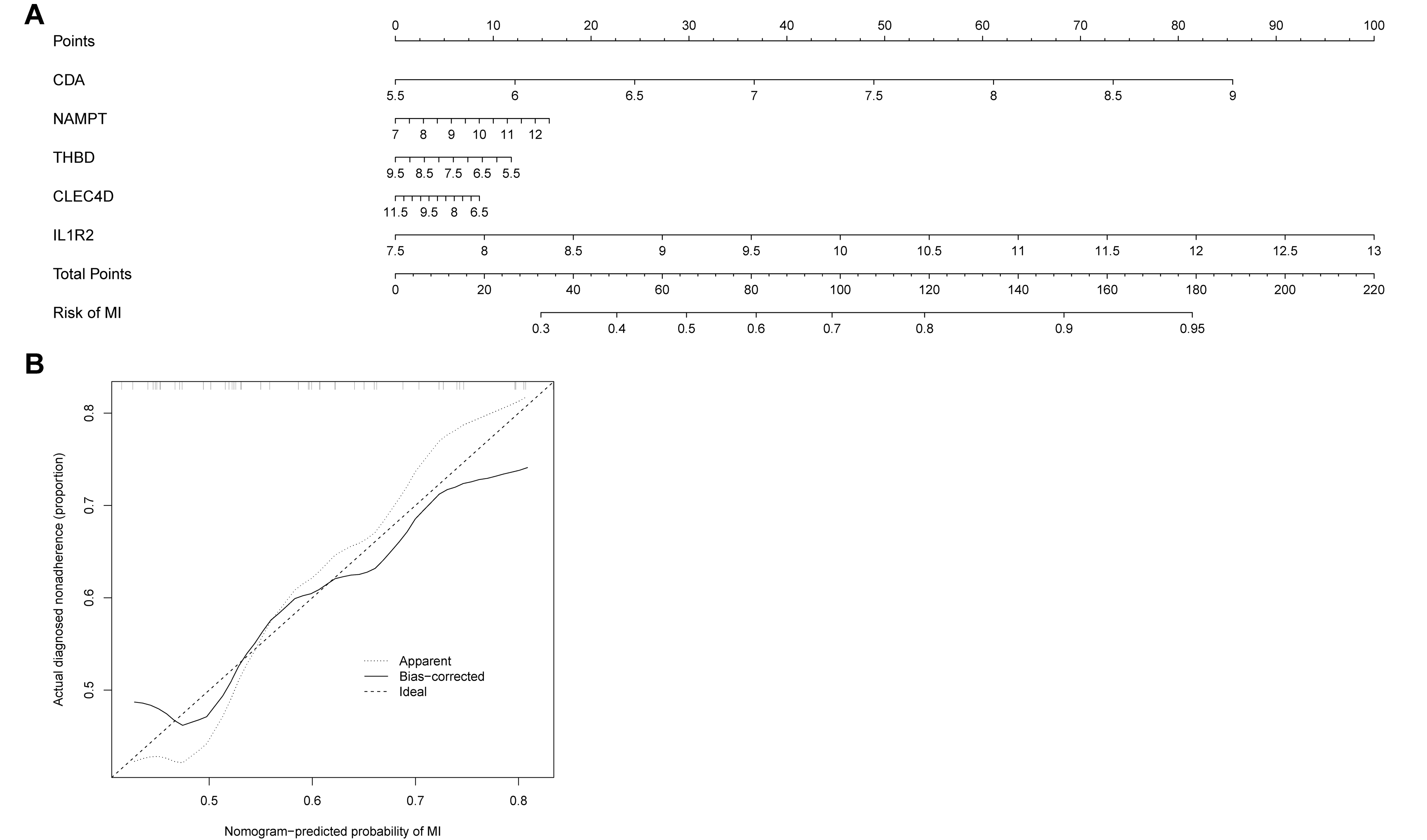


**Figure S5**: Establishment and verification of nomogram based on GSE48060 data. (**A**) A nomogram for predicting risk of suffering from MI and a (**B**) The calibration plot used to validate the nomogram are shown. MI, myocardial infarction.


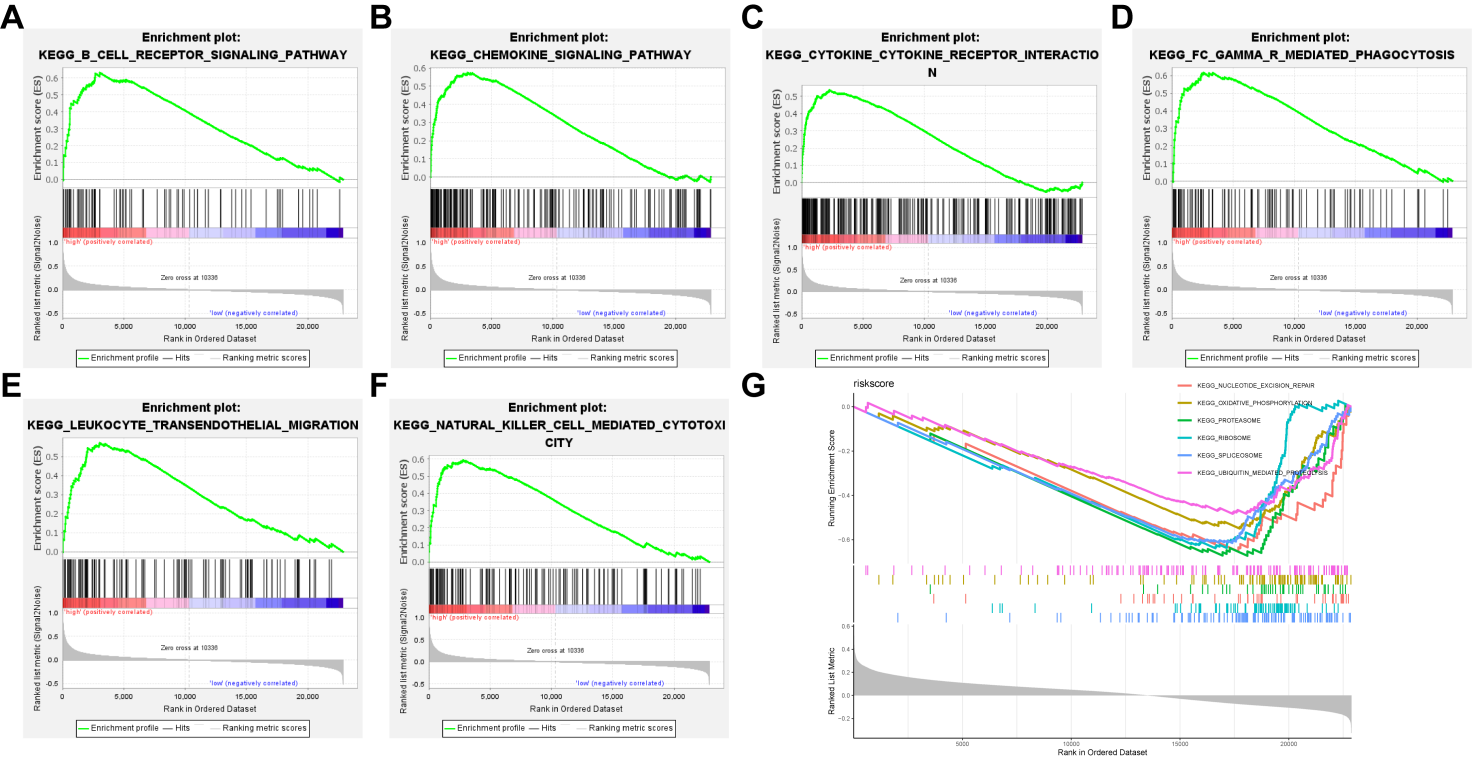


**Figure S6**. GSEA-based assessment of GSE66360 data. (**A**-**F**) GSEA results showing signaling pathway enrichment in those at high risk of suffering from MI. (**G**) A plot revealing signaling pathway enrichment in low-risk MI patients. GSEA, gene set enrichment analysis; MI, myocardial infarction.


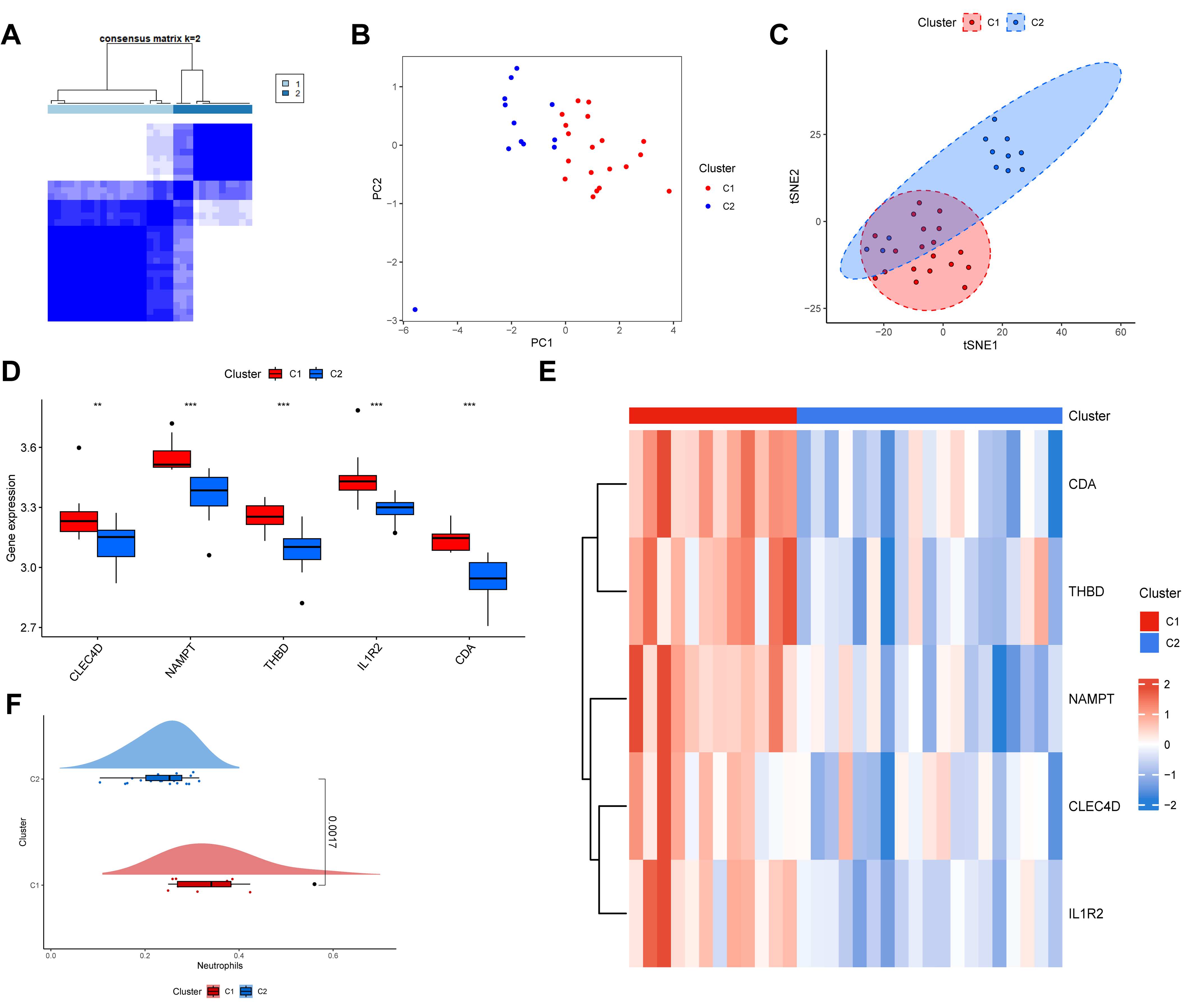


**Figure S7**: Validation of immune clusters via GSE48060 dataset. (**A**) Consensus matrix heatmap shown two clusters of patients. (**B**) PCA and (**C**) t-SNE of the two MI subtypes. (**D**) Boxplot and (**E**) heatmap shown different expression levels of key genes related to neutrophil. (**F**) A violin plot revealed the percentage of infiltrating neutrophils between two clusters. PCA, principal component analysis; t-SNE, t-Distributed stochastic neighbor embedding.


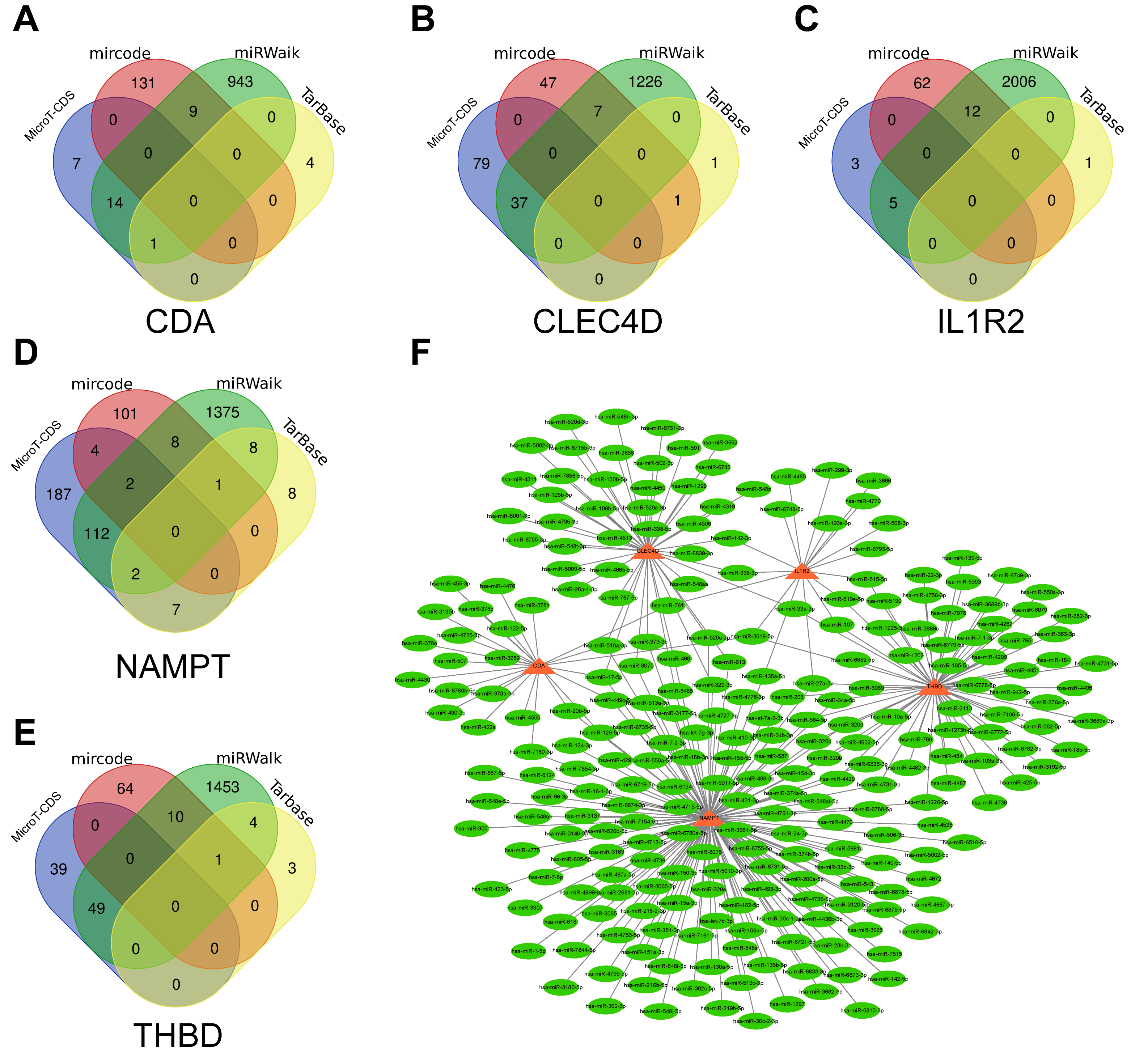


**Figure S8**. Identification of miRNAs and construction of ceRNA network. Results of screens of four databases revealing miRNAs targeting CDA (**A**), CLEC4D (**B**), IL1R2 (**C**), NAMPT (**D**), and THBD (**E**) are shown. (**F**) A ceRNA network shows interactions between our five identified genes and their respective upstream target miRNAs. Red triangles indicate hub genes and green ovals indicate miRNAs. CeRNA network, competitive endogenous RNA network; miRNA, microRNA.


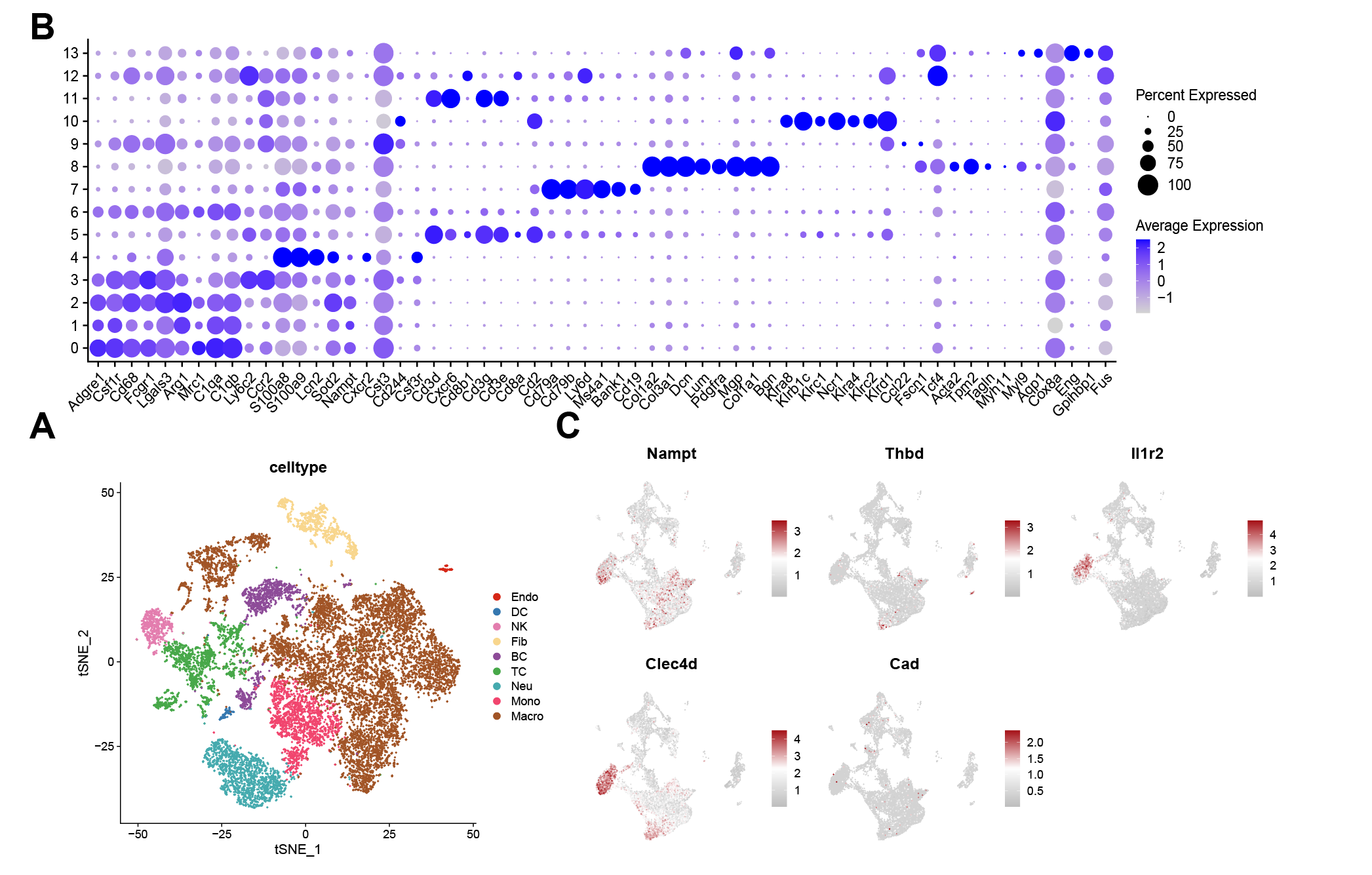


**Figure S9**. ScRNA-seq data from control and infacted heart tissue of mice. (**A**).T-SNE plot shown 9 cell clusters. (**B**) Dot plot of expression of markers in 14 clusters. (**C**) T-SNE plots revealed the expression of hub genes in each cell type. T-SNE, t-Distributed stochastic neighbor embedding.
